# Highly Efficient Purely Organic Phosphorescence Light‐Emitting Diodes Employing a Donor–Acceptor Skeleton with a Phenoxaselenine Donor

**DOI:** 10.1002/advs.202207003

**Published:** 2023-02-20

**Authors:** Zijian Chen, Mengke Li, Qing Gu, Xiaomei Peng, Weidong Qiu, Wentao Xie, Denghui Liu, Yihang Jiao, Kunkun Liu, Jiadong Zhou, Shi‐Jian Su

**Affiliations:** ^1^ State Key Laboratory of Luminescent Materials and Devices and Institute of Polymer Optoelectronic Materials and Devices South China University of Technology Wushan Road 381, Tianhe District Guangzhou Guangdong Province 510640 P. R. China

**Keywords:** organic light‐emitting diode, phenoxaselenine, room‐temperature phosphorescence, single‐molecule white emission

## Abstract

Purely organic room‐temperature phosphorescence (RTP) materials generally exhibit low phosphorescence quantum yield (*ϕ*_P_) and long phosphorescence lifetime (*τ*_P_) due to the theoretically spin‐forbidden triplet state. Herein, by introducing a donor–acceptor (D–A) skeleton with a phenoxaselenine donor, three nonaromatic amine donor containing compounds with high *ϕ*_P_ and short *τ*_P_ in amorphous films are developed. Besides the enhanced spin–orbit coupling (SOC) by the heavy‐atom effect of selenium, the D–A skeleton which facilitates orbital angular momentum change can further boost SOC, and severe nonradiative energy dissipation is also suppressed by the rigid molecular structure. Consequently, a record‐high external quantum efficiency of 19.5% are achieved for the RTP organic light‐emitting diode (OLED) based on 2‐(phenoxaselenin‐3‐yl)‐4,6‐diphenyl‐1,3,5‐triazine (PXSeDRZ). Moreover, voltage‐dependent color‐tunable emission and single‐molecule white emission are also realized. These results shed light on the broad prospects of purely organic phosphorescence materials as highly efficient OLED emitters especially for potential charming lighting applications.

## Graphical abstract

A record‐high external quantum efficiency approaching 20%, voltage‐dependent color‐tunable emissions and single‐molecule white emissions are realized for purely organic electro‐phosphorescence device employing a room‐temperature phosphorescence emitter with a planar donor–acceptor skeleton and a phenoxaselenine donor.

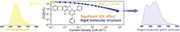

## 1 Introduction

Since the first practical organic light‐emitting diode (OLED) with bilayer heterojunction was reported,^[^ ^1^ ^]^ hypergrowth has been made toward the next‐generation display and lighting technology.^[^ ^2^ ^]^ The luminescent materials used in early OLEDs were conventional fluorescence emitters that could only utilize the spin‐allowed singlet excitons, and the maximum internal quantum efficiency is limited to 25% according to the spin‐statistics. The utilization of the spin‐forbidden triplet excitons has been of vital importance for achieving highly efficient OLEDs. In recent years, phosphorescent materials containing noble heavy metals such as Pt^[^ ^3^ ^]^ and Ir^[^ ^4^ ^]^ have been successfully used as OLED emitters due to their theoretically 100% exciton utilization abilities.^[^ ^5^ ^]^ However, the utilization of noble heavy metals is accompanied by high costs and environmental pollution, so exploiting purely organic phosphorescence materials has recently gained increasing research attention. With effective molecular design concept, such as introducing heavy atom and n–π* transition character to enhance spin–orbit coupling (SOC) and populate the triplet state, room‐temperature phosphorescence (RTP) in purely organic compounds has been successfully realized and extensively explored in the field of anticounterfeiting,^[^ ^6^ ^]^ biological imaging,^[^ ^7^ ^]^ and information encryption.^[^ ^8^ ^]^ Whereas, most of the developed RTP systems are implemented in crystalline state, host–guest system, or polymer matrix, relying on a robust molecular environment to stabilize the triplet excitons and avoid nonradiative transition losses.^[^ ^9^ ^]^ Also, they generally exhibit low phosphorescence quantum yield (*ϕ*_P_) and long phosphorescence lifetime (*τ*_P_), which is not conducive to further OLED application.^[^ ^10^ ^]^ As an essential prerequisite for achieving highly efficient application in OLED devices, developing purely organic RTP materials capable of fully utilizing triplet excitons in amorphous film is still challenging.

Recently, purely organic RTP materials containing aromatic amine fragments have been used as luminescence emitters applied in OLEDs. Nevertheless, there are still many obstacles need to be addressed, such as the low *ϕ*_P_ in amorphous films, which limits the maximum external quantum efficiency (EQE) of devices to around 10%.^[^ ^11^ ^]^ Thus, an effective molecular design strategy is still required to confirm considerable SOC effect to populate the triplet state, and delicate intramolecular interaction manipulation is also indispensable to avoid severe nonradiative energy dissipation induced by molecular motions. Compared with traditional counterparts containing aromatic amines, the chalcogen atoms provide heavy atom effect (expect O) and lone–pair electrons thus enabling the utilization of triplet excitons without the use of precious metals, which is greatly advantageous for achieving highly efficient electroluminescence.^[^ ^11^, ^12^ ^]^ Therefore, incorporating the chalcogen elements and establishing strong intramolecular noncovalent interactions is expected to be one promising strategy for obtaining high *ϕ*_P_ in amorphous films, thus enhancing the performance of corresponding purely organic RTP‐OLEDs.^[^ ^13^ ^]^ On the basis of the above considerations, developing novel organic phosphors capable of fully utilizing triplet excitons in amorphous films is of great significance for achieving highly efficient OLEDs and providing molecular design concepts for the future efficient RTP emitters.

**Figure 1:**
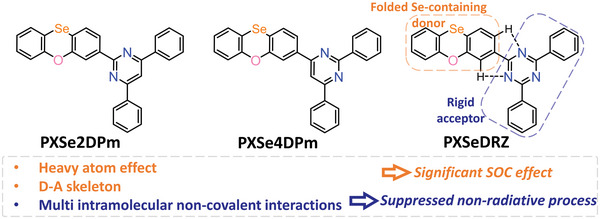
Molecular structures of the investigated compounds and molecular design strategy.

Herein, through connecting nonaromatic amine donor phenoxaselenine (PXSe) with different electron‐deficient fragments in a donor–acceptor (D–A) skeleton, three highly emissive purely organic phosphorescence emitters, namely 2‐(phenoxaselenin‐3‐yl)‐4,6‐diphenyl‐1,3,5‐triazine (PXSeDRZ), 4‐(phenoxaselenin‐3‐yl)‐2,6‐diphenylpyrimidine (PXSe4DPm), and 2‐(phenoxaselenin‐3‐yl)‐4,6‐diphenylpyrimidine (PXSe2DPm) were synthesized (**Figure** **1**; and Figure S1, Supporting Information).^[^ ^14^ ^]^ In addition to achieving enhanced SOC by the heavy‐atom effect of Se, the introduction of D–A skeleton can provide separated frontier molecular orbitals, thus facilitating significant orbital angular momentum change (Δ*L*) that can further boost the singlet–triplet SOC effect. Additionally, the nitrogen atoms of acceptors can establish strong intramolecular noncovalent bond interactions to suppress the severe nonradiative transitions generally caused by the intramolecular vibrations, making these molecular building blocks promising RTP candidates. Consequently, all of them are proved to present high *ϕ*_P_ in amorphous films, and the highest *ϕ*_P_ of 64.0% is achieved for PXSeDRZ with the most effectively suppressed molecular vibrations. A record‐high EQE of 19.5% and nearly unity exciton utilization ratio are achieved for the corresponding purely organic RTP‐OLED. It is noteworthy that all devices show voltage‐dependent tunable color emissions, and single‐molecule white emission could be recorded under high driving voltages. Further theoretical and experimental analyses are thereafter conducted to demonstrate the deeper internal mechanisms for highly efficient purely organic RTP‐OLEDs with such a unique molecular design strategy.

## 2 Results and Discussion

**Figure 2:**
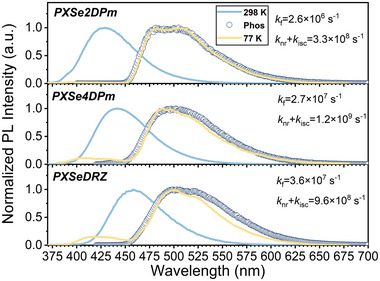
Steady‐state PL (at 298 and 77 K) and phosphorescence (at 77 K with a delay time of 5 ms) spectra of the investigated compounds in toluene solvent (10^−5^ m), and their photophysical parameters of *k*_f_ and *k*_nr_ + *k*_isc_.

Photophysical properties of PXSeDRZ, PXSe4DPm, and PXSe2DPm were investigated both in solvents (10^−5^ m) and in amorphous 10% doped mCP films (Figure S2, Supporting Information). Dissolved in toluene solvent, all of them show intense absorption peaks at ≈280 nm, which are ascribed to the *π*–π* transition. Due to the introduction of D–A skeleton, broad and weak absorption bands are noticed at around 350 nm, which can be assigned to the charge transfer (CT) electronic characters (Figure S3a, Supporting Information). Suffering from intense nonradiative losses induced by dramatic molecular vibrations, steady‐state photoluminescence (PL) spectra reveal that all the three compounds exhibit only fluorescence emission with the lifetime of nanosecond scale at 298 K in toluene solvent (**Figure** **2**; and Figure S3b, Supporting Information).^[^ ^15^ ^]^ With the increase of electron‐withdrawing abilities of acceptors, the observed emission maximum of PXSeDRZ, PXSe4DPm, and PXSe2DPm gradually redshifts from 428 to 440 nm, and to 458 nm. Also, obviously positive solvation effects are observed with increased solvent polarity, further indicating the formation of CT states in these D–A skeletons (Figure S4, Supporting Information). With well‐suppressed nonradiative transition in frozen status, both fluorescence and phosphorescence emissions can be recorded at 77 K, and the phosphorescence emissions dominate the whole PL profile. Also, the photoluminescence quantum yields (*ϕ*_PL_) of PXSeDRZ, PXSe4DPm, and PXSe2DPm in toluene solvent are measured to be 10.6%, 5.6%, and 3.0%, respectively. Based on the measured *ϕ*_PL_ and fluorescence lifetimes, the fluorescence radiative rates (*k*_f_) of all compounds are confirmed to be ≈10^6^ s^−1^, and the combination of nonradiative rates and intersystem crossing (ISC) rates (*k*_nr_ + *k*_isc_) are > 10^8^ s^−1^ (Table S1, Supporting Information). Consequently, the significant larger *k*_nr_ + *k*_isc_ than *k*_f_ can be mainly responsible for their low *ϕ*_PL_ in toluene solvent.

**Figure 3:**
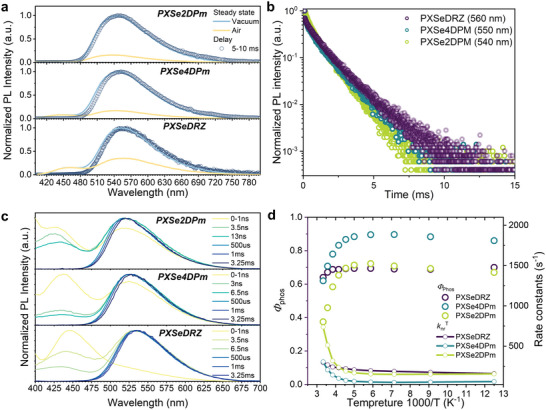
a) Steady‐state PL spectra in air and vacuum status, and phosphorescence spectra in vacuum with a time delay of 5–10 ms. b) Transient phosphorescence decay spectra. c) Time‐resolved PL spectra; d *ϕ*_*P*_ and triplet state non‐radiative rates ($k_{\mathrm{nr}}^{T}$) at different temperatures in vacuum of the investigated compounds in 10 wt% doped mCP films.

In contrast with the individual fluorescence emission in toluene solvent, dual emissions are observed at room temperature when doping these compounds into a universal host 1,3‐bis(*N*‐carbazolyl)benzene (mCP) at a concentration of 10 wt% in amorphous films (**Figure** **3**a). The emission bands at ≈450 nm in the nanosecond lifetime scale correspond well with the fluorescence spectra in toluene solvent and are not sensitive to oxygen, which are ascribed to fluorescence emissions (Figure S5, Supporting Information). The emissions at longer wavelengths of ≈550 nm present mono‐exponential decay characters in the millisecond range and show greatly increased emission intensities in vacuum due to the suppressed triplet exciton quenching by oxygen, which can be attributed to phosphorescence emissions (Figure 3a,b; and Figure S6, Supporting Information). Accordingly, the singlet–triplet splitting energies (Δ*E*_ST_) of PXSeDRZ, PXSe4DPm, and PXSe2DPm are estimated to be 0.77, 0.65, and 0.56 eV, respectively (Table S7, Supporting Information), which are much larger than ambient thermal energy and the possible thermally activated delayed fluorescence process can be excluded.^[^ ^16^ ^]^ In addition, the long‐lived phosphorescence components were further confirmed by temperature‐dependent steady‐state and transient PL decay spectra. As temperature decreases from 300 to 80 K, both the PL intensity and decay lifetime increase due to the suppressed nonradiative channels at low temperatures, which is a representative feature of phosphorescence emission. Besides, the hypochromic phosphorescence spectra at low temperatures can be attributed to the restricted configuration relaxation in frozen state (Figure S7, Supporting Information).^[^ ^10a^ ^]^ Transient PL decay spectra confirm that the *τ*_P_ of PXSeDRZ, PXSe4DPm, and PXSe2DPm are 1.21, 1.24, and 0.85 ms, respectively (Figure 3b). Unlike the low *ϕ*_PL_ caused by the strong nonradiative deactivation of triplet excitons in toluene solvent, the triplet excitons can be effectively utilized in solid state, and the *ϕ*_PL_ of PXSeDRZ, PXSe4DPm, and PXSe2DPm in doped films are measured to be 68.1%, 64.0%, and 38.2%, respectively. Correspondingly, the *ϕ*_P_ are confirmed to be 64.0%, 62.0%, and 37.5% by separating the phosphorescence components from steady‐state PL spectra.

To further explore the origin of the emission, their photophysical properties in doped PMMA films as well as in neat films, and temperature dependent photophysical properties of PXSeDRZ in mesitylene were investigated (Figures S28 and S29, Supporting Information). Taking PXSeDRZ as an example, similar dual emissions of fluorescence at around 450 nm and phosphorescence with single exponential decay in the transient photoluminescence decay curves with millisecond lifetimes at around 560 nm can be observed in neat film, in doped PMMA films with different concentrations and in frozen mesitylene (10^−5^ m) solution, corresponding well with the 10% mCP doped films of the investigated compounds indicating the unchanged dual emission states of fluorescence and phosphorescence (Figure S28a, S28b, Supporting Information). All of them exhibit single exponential decay without prompt component indicating their phosphorescence nature (Figure S28c, S28d, Supporting Information). In dilute solution, only individual fluorescence emission can be observed from 300 to 200 K due to the severe molecular motions can quench their phosphorescence. As the solution frozen under 150 to 80 K, dual emissions of blueshifted fluorescence and phosphorescence can be observed owing to the inhibited triplet nonradiative transitions (Figure S28b, Supporting Information). Increased emission lifetime in millisecond scale can also be observed from 150 to 80 K (Figure S28d, Supporting Information). Note that the singlet emission cannot be totally quenched in solution at room temperature, the totally quenched emission at around 560 nm of PXSeDRZ under room temperature in mesitylene can further exclude the possibility to be singlet emission and provide evidence for the phosphorescence nature.

To explore the detailed exciton dynamic processes of these phenoxaselenine‐based compounds in doped films, time‐resolved emission spectra were measured (Figure 3c). For all compounds, in the initially 1 ns, both the fluorescence and phosphorescence emissions of the emitters can be recorded. And only several nanoseconds later, the phosphorescence emissions at around 550 nm dominate the whole spectra, indicating the extremely efficient ISC processes. After several milliseconds, the phosphorescence spectra exhibit a slight redshift due to the conformation relaxation of triplet excited states. To gain a deeper insight into the exciton dynamics, the detailed rate constants of corresponding exciton dynamic processes were further investigated (**Table** **1**). As expected, all compounds achieve fast intersystem crossing rates (*k*_isc_) of ≈10^8^ s^−1^, which are much faster than their corresponding fluorescence radiative rates (*k*_f_) of ≈10^6^ s^−1^, thus resulting in large intersystem crossing efficiencies (*ϕ*_isc_) of >96% and the dominated phosphorescence emissions at room temperature. Noteworthy, the *k*_isc_ gradually decreases with the increase of Δ*E*_ST_ from PXSeDRZ, PXSe4DPm to PXSe2DPM, whereas the *ϕ*_isc_ is almost unaffected, which can be attributed to the simultaneously decreased *k*_f_.

**Table 1:** Summary of the detailed photophysical parameters of the investigated compounds in 10% doped mCP films

	*ϕ*_PL_ ^a)^	*ϕ*_f_	*ϕ*_P_	*k*_f_ [10^6^ s^−1^]	*k*_isc_ [10^8^ s^−1^]	*k*_P_ [10^2^ s^−1^]	$k_{\mathrm{nr}}^{T}$ [10^2^ s^−1^]	*ϕ*_isc_
PXSeDRZ^b)^	68.1%	4.1%	64.0%	35	8.1	5.3	3.0	96.0%
PXSe4DPm	64.0%	2.0%	62.0%	6.3	3.0	5.0	3.1	98.0%
PXSe2DPm	38.2%	0.7%	37.5%	0.58	0.77	4.4	7.4	99.2%

^a)^ Photoluminescence quantum yields measured in vacuum;

^b)^All the photophysical parameters were calculated according to the Equations (S4)–(S11) in the Supporting Information.

Although all compounds implement an efficient ISC process to populate the triplet state, it is worth noting that they exhibit totally different *ϕ*_P_. To achieve highly efficient phosphorescence emission, the effective suppression of triplet exciton nonradiative dissipation is also an important element. Therefore, exciton kinetic parameters *k*_P_ and $k_{\mathrm{nr}}^{T}$ associated with triplet excitons were calculated to reveal the difference in *ϕ*_P_ (Figure 3d). Results show that all compounds achieve relatively large *k*_P_ of ≈10^2^ s^−1^, which is comparable to their $k_{\mathrm{nr}}^{T}$, indicating the promising molecular design strategy of introducing selenium and the D–A framework. Further, it is noticed that PXSeDRZ and PXSe4DPm achieve higher *k*_P_ than $k_{\mathrm{nr}}^{T}$, thus contributing to their high *ϕ*_P_ of 64.0% and 62.0%, respectively. And $k_{\mathrm{nr}}^{T}$ of PXSe2DPm is larger than its *k*_P_, so that the triplet excitons may face more severe nonradiative deactivation, which accounts for its relatively lower *ϕ*_P_ of 37.5%. In addition, temperature‐dependent exciton kinetic parameters (*ϕ*_P_ and $k_{\mathrm{nr}}^{T}$) were calculated to evaluate the difference in nonradiative transition. As temperature decreases, all compounds exhibit increased *ϕ*_P_ and decreased $k_{\mathrm{nr}}^{T}$ because of restrained molecular motions at low temperatures. However, a completely different changing tendency is noticed for the three compounds (Figure 3d). For PXSe2DPm with low *ϕ*_P_, the $k_{\mathrm{nr}}^{T}$ experiences the most pronounced decrease from 7.4 × 10^2^ s^−1^ at 300 K to 1.6 × 10^2^ s^−1^ at 80 K, thus a more rigid environment is needed to better suppress molecular motions and further improve phosphorescence performance. Noteworthy, PXSeDRZ shows only a slight decrease in $k_{\mathrm{nr}}^{T}$ from 3.0 × 10^2^ s^−1^ at 300 K to 1.6 × 10^2^ s^−1^ at 80 K, indicating a well‐suppressed nonradiative process even at room temperature, which is consistent with its lowest $k_{\mathrm{nr}}^{T}$ among the three compounds.

**Figure 4:**
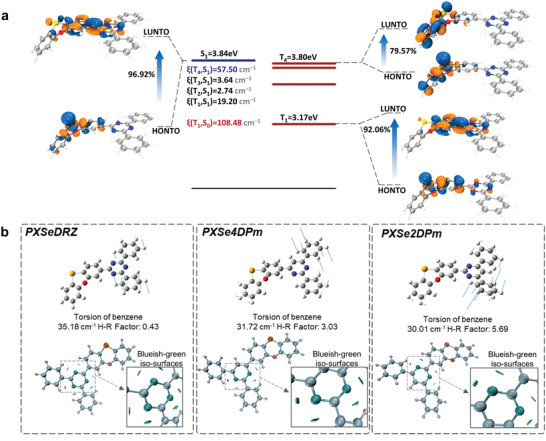
a) Calculated energy level diagram, SOC matrix elements, and NTOs of PXSeDRZ. b) Representative molecular vibration modes and corresponding Huang–Rhys (H–R) factors, and intramolecular interaction visualized by independent gradient model based on Hirshfeld partition (IGMh) analyses.

To gain a deeper insight into the promising RTP performances of the investigated compounds, further theoretical investigations were conducted using density functional theory (DFT) and time‐dependent density functional theory (TD‐DFT) methods.^[^ ^17^ ^]^ First, natural transition orbitals (NTOs) analyses of excited states were carried out to reveal the relationships between molecular structures and electronic characters.^[^ ^18^ ^]^ Taking PXSeDRZ as an example, the energy level diagram of the lowest singlet excited state (S_1_) and triplet excited states (T*_n_*) is presented in **Figure** **4**a. For the energetically close S_1_ and T_4_, different transition orbital characters are observed and an efficient ISC channel may be favored. The highest occupied NTOs (HONTOs) of both S_1_ and T_4_ are similarly located on the PXSe donor, while the distributions of the lowest unoccupied NTOs (LUNTOs) are significantly different. The LUNTO of S_1_ is obviously delocalized to the triazine core of the acceptor and forms a significant long‐range CT state. While the LUNTO of T_4_ is located on the donor, indicating a LE character. According to El‐Sayed's rule, a significant Δ*L* between S_1_ and T_4_ is thus favored to promote spin‐flipping and induce large SOC.^[^ ^19^ ^]^ Cooperated with the heavy atom effect of Se, a large SOC value of 57.50 cm^−1^ is realized between S_1_ and T_4_. Thus, the large SOC and small energy gap between S_1_ and T_4_ significantly contribute to the efficient ISC. In addition, the NTOs of T_1_ were also investigated to explain the fast phosphorescence radiative process. It is noticed that the HONTO of T_1_ is mainly localized on the PXSe donor and the LUNTO is distributed on the triazine acceptor. Further, the PXSe donor exhibits a folded configuration with the lone–pair electron on the selenium, so that the p orbital of the Se atom is not parallel with the orbital on the triazine acceptor, which facilitates Δ*L*.^[^ ^20^ ^]^ Therefore, the combination of large Δ*L* and heavy atom effect ultimately favors the large SOC value of 108.48 cm^−1^ between T_1_ and ground state (S_0_), which contributes to fast *k*_P_.^[^ ^21^ ^]^ Similar transition orbital characters and comparable SOC values are also observed for PXSe4DPm and PXSe2DPm, indicating that the introduction of folded PXSe fragment and D–A framework can boost the SOC effect by heavy atom effect and significant Δ*L* (Figures S10 and S11, Supporting Information). Additionally, the calculated triplet spin density mainly distributes on selenium and central acceptor, which also indicates that both the introduction of selenium and D–A skeleton contribute to the orbital distribution of the triplet state and finally endow them with fast phosphorescence radiative transitions (Figure S12, Supporting Information).

After confirming the efficient ISC and phosphorescence radiative processes, the reorganization energy was further calculated to demonstrate the positive role of introducing a D–A skeleton with rigid acceptor in avoiding severe nonradiative energy dissipation. The calculated reorganization energies between T_1_ and S_0_ ($\lambda_{T_{1}-S_{0}}$) of PXSeDRZ, PXSe4DPm, and PXSe2DPm are 0.710, 0.713, and 0.738 eV, respectively. The small $\lambda_{T_{1}-S_{0}}$ (< 0.8 eV) illustrates a well‐suppressed molecular vibration in these structures and the smallest $\lambda_{T_{1}-S_{0}}$ of PXSeDRZ corresponds well with its experimentally lowest $k_{\mathrm{nr}}^{T}$. Detailed molecular vibration modes were further analyzed by frequency analysis and the corresponding Huang–Rhys factors were calculated using the DUSHIN module in MOMAP program (Figure S13, Supporting Information).^[^ ^22^ ^]^ Results show that the low‐frequency vibration modes with large H–R factors contribute the most to configuration relaxation.^[^ ^23^ ^]^ The representative low‐frequency vibration modes mainly include the torsion of benzene rings on the acceptors, and the out‐of‐plane bending of PXSe donor (Figure 4b; and Figure S14, Supporting Information). And the H–R factor of the benzene torsion vibration mode in PXSeDRZ with the lowest $k_{\mathrm{nr}}^{T}$ is the smallest. As often mentioned, intramolecular interactions may make a significant influence on molecular motion modes. Thereby, the intramolecular interactions were visualized by IGMh analysis to illustrate its effect on the representative vibration modes (Figure 4b).^[^ ^24^ ^]^ According to IGMh, with three nitrogen atoms in the acceptor of PXSeDRZ, there exhibit multi‐intramolecular C—H…N hydrogen bond interactions between the nitrogen atoms and the adjacent hydrogen atoms, which inhibit the severe torsion caused by the attached benzene and suppress nonradiative energy dissipation.^[^ ^25^ ^]^ For PXSe2DPm, a significantly decreased intramolecular interaction is observed owing to the reduced number of nitrogen atoms, resulting in increased molecular motions in contrast to PXSeDRZ, which may account for its relatively larger $k_{\mathrm{nr}}^{T}$. Therefore, by introducing nitrogen atoms in the acceptor to form multiple intramolecular interactions, the resultant D–A skeleton can effectively avoid the non‐radiative losses caused by torsional configuration and improve phosphorescence performance.

**Figure 5:**
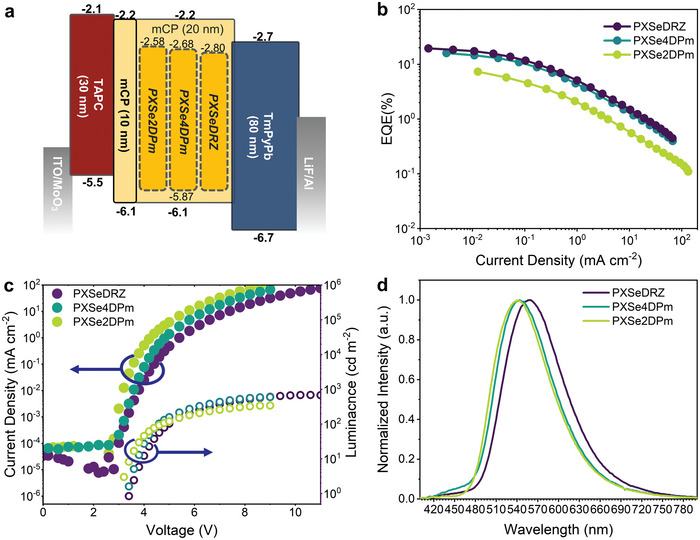
a) Energy‐level diagram and architecture of the OLED devices. b) EQE‐J curves of the OLED devices. c) *J*–*V*–*L* curves of the OLED devices. d) EL spectra of the OLED devices detected at 1 cd m^−2^.

Considering the promising RTP performance in amorphous films, these compounds show great potential for purely organic RTP‐OLEDs. Accordingly, OLED devices were fabricated by employing these compounds as emitters with an architecture shown in **Figure** **5**a, in which indium tin oxide (ITO), molybdenum trioxide (MoO_3_), 4,4’‐cyclohexylidenebis[*N*,*N*‐bis(4‐methylphenyl)benzenamine] (TAPC), 1,3‐bis(*N*‐carbazolyl)benzene (mCP), 1,3,5‐tri(m‐pyrid‐3‐yl‐phenyl)benzene (TmPyPB), lithium fluoride (LiF), and aluminum (Al) serve as anode, hole‐injection, hole‐transporting, hole‐transporting, and electron‐blocking, electron‐transporting, electron‐injection, and cathode materials, respectively.^[^ ^26^ ^]^ The emitting layers were fabricated by doping the emitters into mCP host with a concentration of 10 wt% to avoid severe triplet exciton quenching. The external quantum efficiency‐current density (EQE‐J) and current density–voltage–luminance (*J–V–L*) characteristics are shown in Figure 5a–c. Further detailed device performances are summarized in Table S5 (Supporting Information). The electroluminescent (EL) spectra show that all compounds display almost individual phosphorescence emissions at around 550 nm at low driving voltages (Figure 5d). Also, transient EL decay spectra with mono‐exponential decay characters in the millisecond range further confirm their phosphorescence emission characters (Figure S15b, Supporting Information).^[^ ^27^ ^]^ Owing to their high *ϕ*_P_, maximum EQEs as high as 16.0% and 19.5% are achieved for the devices with PXSe4DPm and PXSeDRZ as emitters, respectively, both representing the highest efficiencies of purely organic phosphorescence OLEDs to date. For the device based on PXSe2PDm as emitter, the relatively low maximum EQE of 7.3% is attributed to its lower *ϕ*_P_ described above. Further, considering a light out‐coupling efficiency of ≈20–30% in conventional OLED devices, nearly unity exciton utilization ratios can be obtained for those devices, indicating that the long‐lived triplet excitons can be effectively utilized for radiation in these purely organic phosphorescence OLEDs. Notably, due to the long‐lived triplet excitons, these devices still suffer severe efficiency roll‐off at high voltages (Figure 5b).^[^ ^28^ ^]^ Since the EQE‐J curves are in good agreement with the triplet–triplet annihilation (TTA) model, the severe efficiency roll‐off of devices can be ascribed to the TTA (Figure S15c, Supporting Information).^[^ ^29^ ^]^ At high voltages, owing to increased triplet excitons density leading to more severe TTA, the intensity of phosphorescence is weakened, while the intensity of fluorescence is enhanced (Figure S16, Supporting Information). As their EL spectra change from individual phosphorescence to the dual emissions of phosphorescence and fluorescence at high voltages, thus tunable emission colors are achieved with increased driving voltages (Figure S17, Supporting Information). Noteworthy, single‐molecule white emissions can be realized for the PXSeDRZ and PXSe4DPm‐based OLEDs with dual emissions at high driving voltages.

## 3 Conclusion

In summary, three compounds with promising RTP performance in amorphous films were synthesized by incorporating Se‐containing phenoxaselenine donor and a donor–acceptor skeleton. Owing to the heavy atom effect combined with significant orbital angular momentum changes induced by the D–A framework, all compounds exhibit greatly enhanced SOC to promote spin‐flipping, thus contributing to their highly efficient ISC and phosphorescence radiative transitions. Moreover, severe nonradiative energy dissipation is greatly suppressed by the rigid molecular structure containing strong noncovalent bond interactions with the PXSe donor. Accordingly, excellent purely organic electro‐phosphorescence with a record‐high EQE approaching 20% was achieved for the first time by employing the developed compounds as emitters. It is also of interest voltage‐dependent color‐tunable emissions and single‐molecule white emissions are realized under high applied voltages for these OLED devices, indicating their charming lighting application potentials. The current work provides a successful molecular design concept for future RTP‐OLEDs and demonstrates the broad prospects of purely organic phosphorescence materials in electroluminescence.

Notably, apart from realizing highly efficient RTP‐OLEDs, severe issues remain unaddressed for the serious efficiency roll‐off, low maximum luminance and unstable EL spectra of the currently developed RTP‐OLEDs. Other than high phosphorescence quantum yield in amorphous film, further reduction of phosphorescence lifetime for rapid phosphorescence radiation to avoid triplet quenching and increment of the ISC rates to fully convert singlet excitons into triplets are critical. Further exploration of novel phenoxaselenine‐containing materials is needed to further improve the RTP‐OLED performance.

## 4 Experimental Section

### Chemical Structure Characterization

^1^H and ^13^C NMR spectra were recorded on a Bruker NMR spectrometer operating at 500 and 126 MHz, respectively. The samples were dissolved in deuterated chloroform (CDCl_3_) or (Methyl sulfoxide)‐d6 (DMSO‐*d*_6_) and measured at ambient temperature. High resolved mass spectra (HRMS) were obtained using a Bruker maXis impact mass spectrometer. Differential scanning calorimetry (DSC) measurements were performed on Netzsch DSC 209 under nitrogen flow at two heating and cooling cycles with a heating rate of 10 °C min^−1^ and a cooling rate of 20 °C min^−1^. Thermogravimetric analyses (TGA) were performed on Netzsch TG 209 under nitrogen flow at a heating rate of 10 °C min^−1^. For single crystal X‐ray analysis, the data were collected on Rigaku XtaLAB P2000 FR‐X with a rotating copper anode and a Pilatus 200K detector at room temperature. The corresponding data (PXSeDRZ: CCDC 2 194 114, PXSe4DPm: CCDC 2 194 116, and PXSe2DPm: CCDC 2 194 115) can be obtained free of charge from the Cambridge Crystallographic Data Centre. High performance liquid chromatography spectra were measured using Waters alliance e2695 separation module to grantee the purity of the investigated compounds (Figure S40, Supporting Information).

### Quantum Chemistry Simulation

The geometry optimizations and excited‐state properties simulations were performed using the Gaussian 09 E01 package.^[^ ^30^ ^]^ All S_0_ geometries were optimized in m06‐2x/def2‐SVP level in the gas phase according to the DFT.^[^ ^31^ ^]^ The *S*_1_ geometries were optimized in the m06‐2x/def2‐SVP level in the gas phase according to the TD‐DFT, while the *T*_1_ geometries were optimized using m06‐2x/def2‐SVP in the gas phase according to the unrestricted DFT.^[^ ^17^ ^]^ Further frequency analyses were calculated in geometries optimization to accurately find the local minima. Based on the optimized geometries, excited state properties were subsequently investigated using TD‐DFT in the m06‐2x/def2‐TZVP level. The NTOs and IGMh^[^ ^24^ ^]^ were analyzed using Mutiwfn^[^ ^18^ ^]^ and visualized with VMD software.^[^ ^32^ ^]^ The corresponding SOC matrix elements were calculated using ORCA based on the optimized geometries in m06‐2x/def2‐TZVP.^[^ ^33^ ^]^ The H‐R factors were determined with the DUSHIN module in MOMAP (Molecular Materials Property Prediction Package).^[^ ^22b‐e^ ^]^

### Photophysical Characterization

UV–vis absorption spectra were recorded using the Perkin‐Elmer Lambda 950‐PKA instrument. Fluorescence and phosphorescence spectra were recorded by a FluoroMax‐4 spectrofluorometer. Transient PL decay spectra were conducted on FLS980 (Edinburgh Instrument), in which the prompt component in the nanosecond scale was measured with a TCSPC laser and the delayed component in microsecond scales was measured with a microsecond flash lamp. Their delayed spectra were measured in FLS980 utilizing a time‐gated controller. Temperature‐dependent PL spectra and transient PL decay spectra of the film samples were also measured on FLS980 equipped with Oxford Instruments nitrogen cryostat (Optistat DN) for temperature control. PLQYs of the films were measured on FLS980 utilizing an integrating sphere, while the PLQYs of the solutions were measured utilizing a QE 2000 quantum efficiency measurement system (Otsuka Electronics). The singlet and triplet energies were calculated using the onset of the emissions (Figure S21, Supporting Information).

### Device Fabrication and Characterization

All the organic materials for the device fabrication were available from commercial sources. The corresponding energy levels of organic semiconductor films were obtained from photoelectron yield measurement (AC‐3). Before device fabrication, Indium‐tin oxide (ITO) coated glass substrates were washed with ultrasonic sequentially in deionized water, acetone, and ethanol and treated with O_2_ plasma for 2 min before fabrication. The organic layers were deposited onto the substrate in a vacuum chamber under a high vacuum (<10^−4^ Pa). The deposition rates of the organic materials, LiF, and Al were ≈0.8–1.2, 0.1, and 3 Å s^−1^, respectively. The current density and luminance versus driving voltage characteristics and EL spectra were recorded by an optical analyzer, Photo Research PR745, and powered by Keithley 2400. External quantum efficiency was calculated from the luminance, current density, and EL spectrum, assuming a Lambertian distribution. The size of the pixels is 0.9 cm^2^. The transient EL measurements and delayed EL spectra were conducted on an FL980 spectrometer (Edinburg Instrument) with a time‐gated controller. The pulse voltages were generated by an oscilloscope (RIGOLDG4162), with a period of 20 ms and a plus width of 2 ms for the measurement. The HOMO energy levels were estimated from the photoelectron yield measurement of the neat film. The LUMO energy levels were calculated by HOMO energy levels minus optical energy gaps. And the optical energy gaps were reckoned from the onset of the absorption curve of the investigated compounds in toluene solvent (10^−5^ m) (Figure S20, Supporting Information).

## Conflict of Interest

The authors declare no conflict of interest.

## Supporting information

Supporting Information

## Data Availability

The data that support the findings of this study are available from the corresponding author upon reasonable request.
