# Supplementary material for: Highly Efficient Purely Organic Phosphorescence Light‐Emitting Diodes Employing a Donor–Acceptor Skeleton with a Phenoxaselenine Donor
Source: Adv Sci (Weinh). 2023 Feb 20;10(12):2207003. doi: 10.1002/advs.202207003 (PMC10131844; doi:10.1002/advs.202207003)
Supplement: Supplementary file 1 — Supporting Information [file ADVS-10-2207003-s001.pdf]

ADVANCED SCIENCE
Open Access

Supporting Information

for *Adv. Sci.*, DOI 10.1002/advs.202207003

Highly Efficient Purely Organic Phosphorescence Light-Emitting Diodes Employing a Donor–Acceptor Skeleton with a Phenoxaselenine Donor

*Zijian Chen, Mengke Li, Qing Gu, Xiaomei Peng, Weidong Qiu, Wentao Xie, Denghui Liu, Yihang Jiao, Kunkun Liu, Jiadong Zhou and Shi-Jian Su**

Supporting Information

Highly Efficient Purely Organic Phosphorescence Light-Emitting Diodes Employing a Donor-Acceptor Skeleton with a Phenoxaselenine Donor

Zijian Chen,[†] Mengke Li,[†] Qing Gu, Xiaomei Peng, Weidong Qiu, Wentao Xie, Denghui Liu, Yihang Jiao, Kunkun Liu, Jiadong Zhou & Shi-Jian Su*

[*] Z. Chen, Dr. M. Li, Q. Gu, X. Peng, W. Qiu, Dr. W. Xie, D. Liu, Y. Jiao, Dr. K. Liu, Dr. J. Zhou, Prof. S.-J. Su
State Key Laboratory of Luminescent Materials and Devices and Institute of Polymer Optoelectronic Materials and Devices, South China University of Technology
Wushan Road 381, Tianhe District, Guangzhou 510640, Guangdong Province (P. R. China)
E-mail: mssjsu@scut.edu.cn
[†] These authors contributed equally to this work.

Table of Contents

1 Synthesis

2 Results and Discussion

2.1 Evaluation of exciton dynamic rate constants

2.2 Photophysical properties

2.3 Single crystals information

2.4 Theoretical calculations

2.5 Device characterizations

2.6 Energy levels

2.7 Identification of the EL emission in OLED devices

2.8 Further identification of the origin of the emissions

3 Figures of the chemical structure characterizations

4 Reference

5 Author Contributions

6 Molecular Coordinates

1 Synthesis

PXSe2DPm, PXSe4DPm and PXSeDRZ were synthesized according to the synthetic routes descried in Figure S1.[1] Detailed synthetic routes, procedures and basic characterization of the target compounds are given below.

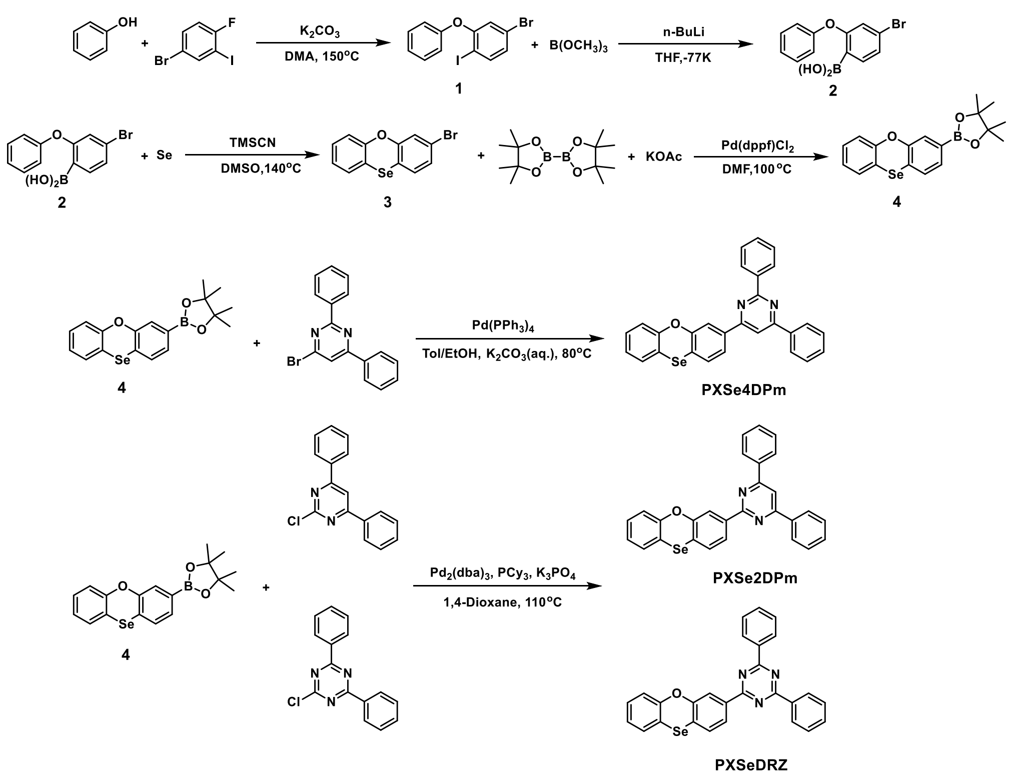

Figure S1. Synthetic routes. Detailed synthetic routes of the investigated compounds.

Synthesis of compound 4-bromo-1-iodo-2-phenoxybenzene (1). Phenol (51.2 mmol, 4.82 g), 4-bromo-1-fluoro-2-iodobenzene (39.4 mmol,11.86 g) and K_2CO_3 were dissolved into 100 mL dimethylacetamide in a 250mL round-bottom three-neck flask. Under a nitrogen atmosphere, the reaction mixture was then heated to 150℃ and stirred vigorously for 24 hours. After the reaction system was cooled to ambient temperature, the solvent was removed in a vacuum and the residues were extracted with dichloromethane. Further column chromatography using petroleum ether as the eluent affords 11.89 g colorless solids (80.5% yield). ^{1}H NMR (500 MHz, Chloroform-d) δ 7.47 (d, J = 8.5 Hz, 1H), 7.40 – 7.34 (m, 2H), 7.16 (tt, J = 7.4, 1.1 Hz, 1H), 7.12 (dd, J = 8.5, 2.3 Hz, 1H), 7.04 – 6.97 (m, 3H).

Synthesis of compound (4-bromo-2-phenoxyphenyl)boronic acid (2). 4-bromo-1-iodo-2-phenoxybenzene (26.67 mmol,10.00 g) was dissolved into 100 mL tetrahydrofuran in a 250 mL round bottom three-neck flask. The reaction mixture was cooled to -78 ℃ for 15 minutes. Under a nitrogen atmosphere, 1.6 M n-butyllithium (28 mmol, 17.5 mL) dissolved in n-Hexane was added dropwise into the reaction mixture. And the reaction was stirred vigorously for 1 hour. Then, the reaction mixture was allowed to return to room temperature and stirred for another 12 hours. After that, 100 mL H_2O was added to quench the reaction. And the resulting mixture was washed with H_2O three times and extracted with dichloromethane. Further column chromatography using dichloromethane as the eluents affords 2.74 g white solid (35.1% yields). ^{1}H NMR (500 MHz, Chloroform-*d*) δ 7.79 (d, *J* = 8.0 Hz, 1H), 7.49 – 7.36 (m, 2H), 7.26 (ddt, *J* = 7.8, 4.4, 1.9 Hz, 2H), 7.13 – 7.06 (m, 2H), 6.83 (d, *J* = 1.7 Hz, 1H), 6.01 (s, 2H).

Synthesis of compound 3-bromophenoxaselenine (3). (4-bromo-2-phenoxyphenyl)boronic acid (6.83 mmol,2.02 g), Trimethylsilyl cyanide (0.20 mmol, 20 mg) and selenium (20.49 mmol, 1.62 g) was dissolved into 50mL Dimethyl sulfoxide in a 100 mL round bottom three-neck flask. The reaction mixture was heated to 140 ℃ for 24 hours. After the reaction system was cooled to ambient temperature, the resulting mixture was washed with H_2O three times and extracted with dichloromethane. Further column chromatography using petroleum ether affords 1.92 g white solid (86.3% yields). ^{1}H NMR (500 MHz, Chloroform-*d*) δ 7.26 (dd, *J* = 9.4, 2.4 Hz, 2H), 7.22 – 7.11 (m, 3H), 7.09 (dd, *J* = 8.1, 1.4 Hz, 1H), 7.04 (td, *J* = 7.5, 1.4 Hz, 1H).

Synthesis of compound 4,4,5,5-tetramethyl-2-(phenoxaselenin-3-yl)-1,3,2-dioxaborolane (4). 3-bromophenoxaselenine (3.07 mmol, 1.00 g), 4,4,4',4',5,5,5',5'-octamethyl-2,2'-bi(1,3,2-dioxaborolane) (9.21 mmol, 2.34 g) and potassium acetate (9.21 mmol, 0.90 g) were dissolved into 50mL N,N-Dimethylformamide in a 100 mL round bottom three-neck flask Under a nitrogen atmosphere, 1,1'-Bis(diphenylphosphino)ferrocene]dichloropalladium(II) (0.15 mmol, 112mg) was added into the flask. The reaction mixture was then heated to 100℃, stirred vigorously for 12 hours. After the reaction system was cooled to ambient temperature, the resulting mixture was washed with H_2O three times and extracted with dichloromethane. Further column chromatography using petroleum ether/dichloromethane in 2/1 volume as the eluent affords 778 mg white solid (67.9% yields). ^{1}H NMR (500 MHz, DMSO-d_6) δ 7.54 – 7.44 (m, 2H), 7.39 – 7.32 (m, 2H), 7.28 (ddd, *J* = 8.1, 7.3, 1.6 Hz, 1H), 7.19 (dd, *J* = 8.1, 1.4 Hz, 1H), 7.12 (td, *J* = 7.5, 1.4 Hz, 1H), 1.29 (s, 12H).

Synthesis of compound 4-(phenoxaselenin-3-yl)-2,6-diphenylpyrimidine (PXSe4DPm). 4,4,5,5-tetramethyl-2-(phenoxaselenin-3-yl)-1,3,2-dioxaborolane (4.4 mmol, 1.67 g), 4-bromo-2,6-diphenylpyrimidine (6.7 mmol, 2.1 g), K_2CO_3 (2 M in H_2O, 40 mL), toluene (120 mL) and ethanol (50 mL) were added into a 250 mL three-necked flask under a argon atmosphere. After stirring vigorously at room temperature for 20 min, tetrakis(triphenyl-phosphine) palladium(0) (Pd(PPh3)4) (254 mg, 0.05eq) was added into the flask. The reaction mixture was then heated to 80℃, stirred vigorously for 12 hours. After the reaction system was cooling to ambient temperature, the solvent was removed in a vacuum and the residues were extracted with dichloromethane. Further column chromatography using petroleum ether/dichloromethane in 5/1 volume as the eluent affords 1.10 g yellow solids (52.3% yield). ^{1}H NMR (500 MHz, Chloroform-*d*) δ 8.74 – 8.66 (m, 2H), 8.28 (dd, *J* = 7.6, 2.0 Hz, 2H), 8.05 (d, *J* = 1.9 Hz, 1H), 7.99 – 7.92 (m, 2H), 7.60 – 7.49 (m, 6H), 7.45 (d, *J* = 8.0 Hz, 1H), 7.31 (dd, *J* = 7.7, 1.5 Hz, 1H), 7.24 – 7.15 (m, 2H), 7.07 (td, *J* = 7.3, 1.6 Hz, 1H). ^{13}C NMR (126 MHz, $CDCl_3$) δ 164.94, 164.56, 163.36, 153.45, 152.71, 137.99, 137.93, 137.39, 130.91, 130.77, 129.72, 129.39, 128.96, 128.54, 128.50, 127.33, 125.27, 123.57, 119.93, 118.87, 117.12, 115.55, 109.86, 77.36, 77.04, 76.72. HRMS (ESI$^+$): calculated for $C_{28}H_{19}N_2OSe$ [M]$^+$ 479.0659, found 479.0663.

Synthesis of compound 2-(phenoxaselenin-3-yl)-4,6-diphenylpyrimidine (PXSe2DPm). 4,4,5,5-tetramethyl-2-(phenoxaselenin-3-yl)-1,3,2-dioxaborolane (3.00 mml, 1.12 g), 2-chloro-4,6-diphenylpyrimidine (6.00 mml, 1.60 g) and K_3PO_4 (14.60 mmol, 3.10 g) were dissolved into 100 mL 1,4-dioxane in a 250 mL round-bottom three-neck flask. Under nitrogen atmosphere, tricyclohexylphosphine (0.31 mmol, 86 mg), tris(dibenzylideneacetone)dipalladium (0.192 mmol, 176 mg) were added into the flask. The reaction mixture was then heated to 110 ℃, stirred vigorously for another 18 hours. After the reaction system was cooling to ambient temperature, the solvent was removed in a vacuum and the residues were extracted with dichloromethane. Further column chromatography using petroleum ether/dichloromethane in 5/1 volume as the eluent affords 328 mg yellow solids (22.9% yield). ^{1}H NMR (500 MHz, Chloroform-*d*) δ 8.46 (d, *J* = 1.8 Hz, 1H), 8.41 (dd, *J* = 8.1, 1.8 Hz, 1H), 8.28 (dd, *J* = 7.8, 1.8 Hz, 4H), 8.01 (s, 1H), 7.59 – 7.53 (m, 6H), 7.43 (d, *J* = 8.1 Hz, 1H), 7.31 (dd, *J* = 7.6, 1.4 Hz, 1H), 7.22 (dd, *J* = 6.5, 1.7 Hz, 2H), 7.06 (ddd, *J* = 7.7, 6.3, 2.3 Hz, 1H). ^{13}C NMR (126 MHz, $CDCl_3$) δ 164.83, 163.45, 153.22, 152.99, 138.79, 137.36, 130.92, 129.38, 129.27, 128.97, 128.42, 127.35, 125.08, 124.99, 119.66, 118.92, 118.34, 115.93, 110.58, 77.36, 77.04, 76.73. HRMS (ESI$^+$): calculated for $C_{28}H_{19}N_2OSe$ [M]$^+$ 479.0659, found 479.0666.

Synthesis of compound 2-(phenoxaselenin-3-yl)-4,6-diphenyl-1,3,5-triazine (PXSeDRZ). 4,4,5,5-tetramethyl-2-(phenoxaselenin-3-yl)-1,3,2-dioxaborolane (3.00 mml, 1.12 g), 2-chloro-4,6-diphenyl-1,3,5-triazine (6.00 mmol, 1.61 g) and K_3PO_4 (14.60 mmol, 3.10 g) were dissolved into 100 mL 1,4-dioxane in a 250mL round-bottom three-neck flask. Under nitrogen atmosphere, tricyclohexylphosphine (0.31 mmol, 86 mg), tris(dibenzylideneacetone)dipalladium (0.192 mmol, 176 mg) were added into the flask. The reaction mixture was then heated to 110 ℃, stirred vigorously for another 18 hours. After the reaction system was cooling to ambient temperature, the solvent was removed in a vacuum and the residues were extracted with dichloromethane. Further column chromatography using petroleum ether/dichloromethane in 5/1 volume as the eluent affords 292 mg yellow solids (20.4% yield). ^{1}H NMR (500 MHz, Chloroform-*d*) δ 8.78 – 8.71 (m, 4H), 8.47 – 8.37 (m, 2H), 7.58 (dddd, *J* = 14.5, 8.5, 6.1, 2.2 Hz, 6H), 7.44 (d, *J* = 8.0 Hz, 1H), 7.30 (dd, *J* = 7.6, 1.4 Hz, 1H), 7.25 – 7.18 (m, 2H), 7.07 (ddd, *J* = 7.7, 6.6, 2.0 Hz, 1H). ^{13}C NMR (126 MHz, $CDCl_3$) δ 171.70, 170.69, 153.22, 152.73, 136.84, 136.11, 132.61, 129.45, 129.35, 129.02, 128.67, 128.58, 128.26, 125.31, 125.25, 122.29, 118.92, 118.56, 115.50, 77.35, 77.03, 76.72, 0.02. HRMS (ESI$^+$): calculated for $C_{27}H_{18}N_3OSe$ [M]$^+$ 480.0611, found 480.0617.

2 Results and Discussion

2.1 Evaluation of exciton dynamic rate constants

In solution, there is only a prompt component of fluorescence. The lifetime of the prompt emission (τ_{prompt}) depends on the rate constants of fluorescence (k_f.), non-radiative transition (k_{nr}), and intersystem crossing (k_{ISC}):

$\tau_{prompt} = \frac{1}{k_f+k_{nr}+k_{ISC}}$ (Equation S1)

Therefore, the k_f and $k_{nr} + k_{ISC}$ in the solution are calculated as:

$k_f = \frac{\Phi_{PL}}{\tau_{prompt}}$ (Equation S2)

$k_{nr} + k_{ISC} = \frac{1}{\tau_{prompt}} - k_f$ (Equation S3)

In the film state, there are dual emissions of fluorescence and phosphorescence dominated by phosphorescence. The quantum yields of fluorescence (Φ_f) and phosphorescence (Φ_P) were separated using the steady-state PL spectra and delayed PL spectra. The photoluminescence quantum yield (Φ_{PL})can be expressed as:

$\Phi_{PL} = \Phi_P + \Phi_f$ (Equation S4)

Because of the dominated phosphorescence emission and high phosphorescence quantum yields, we assume that the non-radiative transition of singlet can be ignored, thus the fluorescence lifetime (τ_f) can be expressed as:

$\tau_f = \frac{1}{k_f+k_{ISC}}$ (Equation S5)

Therefore, the k_f and k_{ISC} in the film state is calculated as:

$k_f = \frac{\Phi_f}{\tau_f}$ (Equation S6)

$k_{ISC} = \frac{1}{\tau_f} - k_f$ (Equation S7)

Under photo-excited, the generated singlet excitons deactivate thought fluorescence emission and intersystem crossing, thus the intersystem crossing efficiency (Φ_{ISC}) can be expressed as:

$\Phi_{ISC} = 1 - \Phi_f$ (Equation S8)

After intersystem crossing, the generated triplet excitons are deactivated through phosphorescence radiative and triplet non-radiative transition, thus the phosphorescence lifetime (τ_P) can be expressed as:

$\tau_P = \frac{1}{k_P+k_{nr}^T}$ (Equation S9)

Therefore, the rate constants of phosphorescence (k_P) and non-radiative transition of triplet (k_{nr}^T) in the film state can be expressed as:

$k_P = \frac{\Phi_P}{\tau_P}$ (Equation S10)

$k_{nr}^T = \frac{1}{\tau_P} - k_P$ (Equation S11)

2.2 Photophysical properties

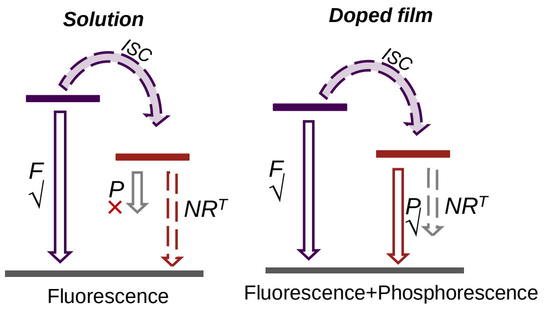

Figure S2. Scheme of the photophysical processes in toluene solvent and 10% doped mCP films at 298 K. (F: fluorescence, P: phosphorescence and NR^T: triplet non-radiative process).

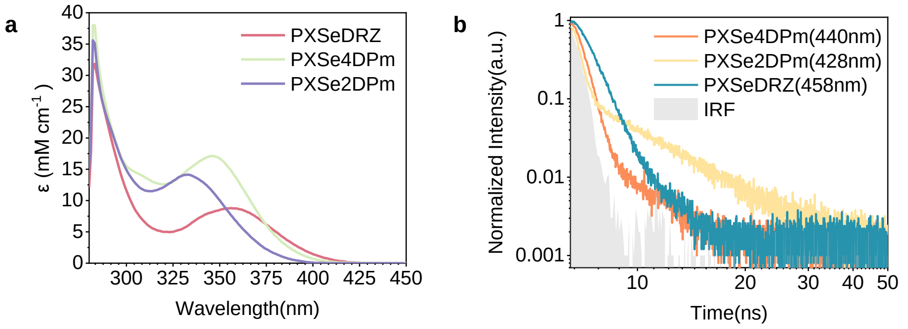

Figure S3. a UV-vis absorption and **b** transient PL decay spectra of PXSeDRZ, PXSe4DPm, and PXSe2DPm in toluene solvent (10^{-5} M) at 298K.

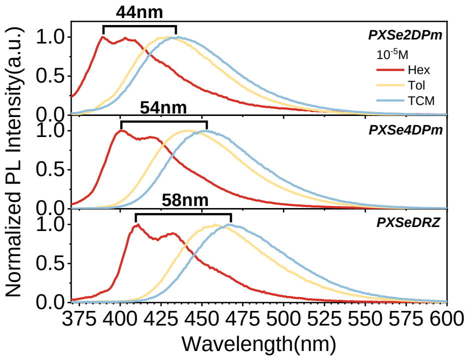

Figure S4. PL spectra of PXSe2DPm, PXSe4DPm, and PXSeDRZ in n-hexane, toluene, and trichloromethane solvents (10^{-5} M).

Table S1. Summary of the photophysical properties of the investigated compounds in toluene solvent.

	In toluene Solvent (10^{-5} M)[a]		
	Φ_{PL}	k_f [10^7 s^{-1}]	$k_{nr}+k_{ISC}$ [10^8 s^{-1}]
PXSeDRZ	10.6%	3.6	9.6
PXSe4DPm	5.6%	2.7	12
PXSe2DPm	3.0%	0.26	3.3

[a] All of the photophysical properties were calculated using Equation S1-S4.

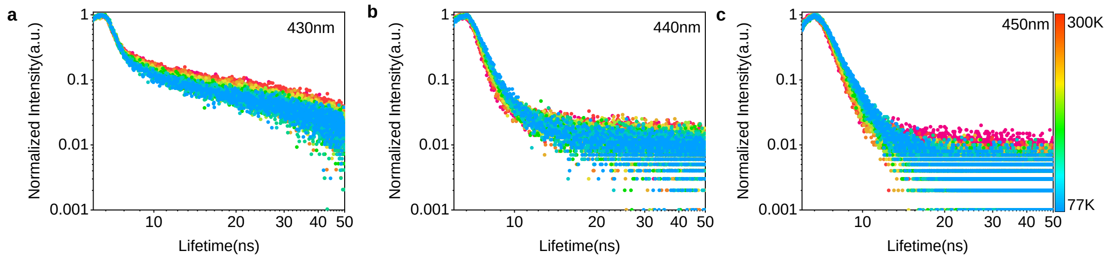

Figure S5. Temperature-dependent Transient PL decay spectra of **a** PXSe2DPm, **b** PXSe4DPm and **c** PXSeDRZ in 10% doped mCP films.

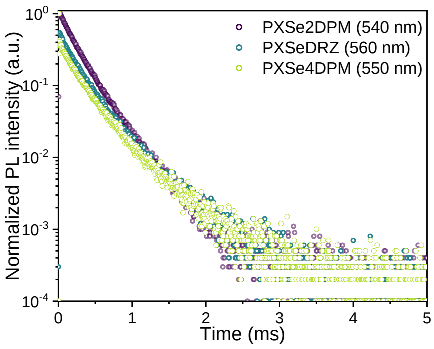

Figure S6. Transient phosphorescence decay spectra in air condition of the three investigated compounds in 10% doped mCP films.

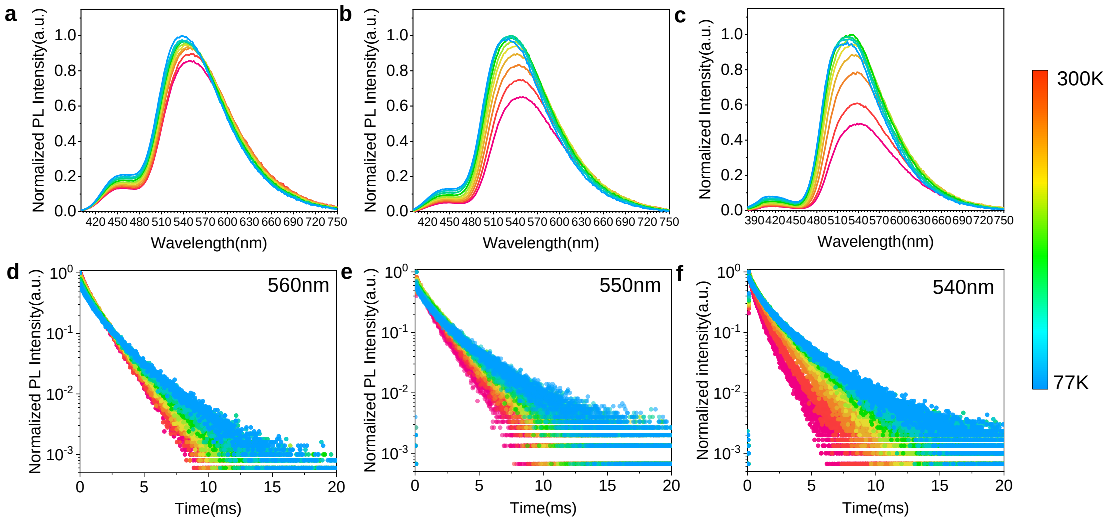

Figure S7. Temperature-dependent PL spectra of **a** PXSeDRZ, **b** PXSe4DPm and **c** PXSe2DPm in 10% doped mCP films. Temperature-dependent transient PL decay spectra of **d** PXSeDRZ, **e** PXSe4DPm and **f** PXSe2DPm in 10% doped mCP films.

Table S2. Summary of the corresponding fluorescence and phosphorescence lifetimes of all the three compounds in toluene solvent and 10% doped mCP films.

	Solution	Doped film	
	τ_{prompt}[ns]	τ_f[ns]	τ_P[ms]
PXSeDRZ	0.92	1.18	1.21
PXSe4DPm	0.80	3.21	1.24
PXSe2DPm	2.95	12.86	0.85

Table S3. Summary of the phosphorescence quantum yields (ϕ_P) and triplet state non-radiative rates (k_{nr}^T) at different temperatures in vacuum of the investigated compounds in 10 wt.% doped mCP films.

	ϕ_P(%)			k_{nr}^T(s^{-1})		
	PXSeDRZ	PXSe4DPm	PXSe2DPm	PXSeDRZ	PXSe4DPm	PXSe2DPm
300 K	64.0	62.0	37.5	297.2	305.6	737.9
280 K	67.1	70.7	45.9	268.5	206.5	512.1
260 K	68.8	78.0	58.4	241.2	142.4	318.7
240 K	68.8	83.2	65.3	230.9	100.9	235.6
220 K	69.2	86.7	69.5	221.9	76.0	190.5
200K	69.4	88.4	71.4	207.4	63.4	168.3
170 K	69.4	89.6	72.3	196.4	54.7	153.9
140 K	69.0	89.7	70.9	187.0	50.5	150.2
110 K	68.8	88.3	69.3	176.0	54.1	152.0
80 K	70.0	86.0	67.0	159.0	60.2	155.5

2.3 Single crystals information

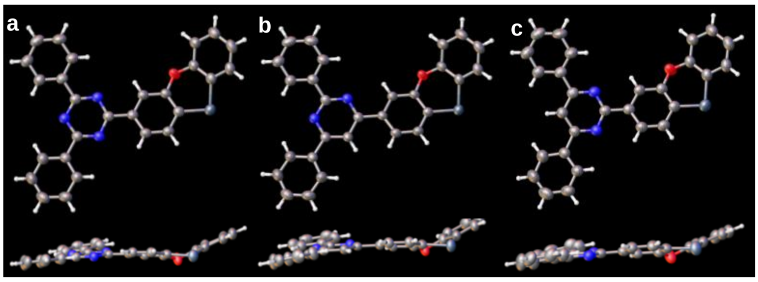

Figure S8. Front and side views of molecular geometries obtained from the single crystal X-ray analysis: **a** PXSeDRZ, **b** PXSe4DPm and **c** PXSe2DPm.

Table S4. Crystal data and structure refinement for PXSeDRZ, PXSe4DPm and PXSe2DPm.

Identification code	PXSeDRZ (CCDC 2194114)	PXSe4DPm (CCDC 2194116)	PXSe2DPm (CCDC 2194115)
Empirical formula	$C_{27}H_{17}N_3OSe$	$C_{27}H_{18}N_2OSe$	$C_{27}H_{18}N_2OSe$
Formula weight	478.39	477.40	477.40
Temperature/K	301.42(10)	301.39(13)	301.22(10)
Crystal system	orthorhombic	orthorhombic	orthorhombic
Space group	$Pna2_1$	$Pna2_1$	$Pna2_1$
a/Å	6.64295(15)	16.50345(19)	16.4743(3)
b/Å	6.12587(6)	6.14967(7)	6.25159(10)
c/Å	20.69900(20)	20.9296(2)	20.7528(4)
α/°	90	90	90
β/°	90	90	90
γ/°	90	90	90
Volume/Å^3	2110.32	2124.16	2137.34
Z	4	4	4
Radiation	CuKα(λ=1.54184)	CuKα(λ=1.54184)	CuKα(λ=1.54184)
R indices [I>2sigma(I)]	R1=0.0217 wR2=0.0596	R1=0.0278 wR2=0.0767	R1=0.0275 wR2=0.0683
R indices (all data)	R1=0.0221 wR2=0.0599	R1=0.0282 wR2=0.0769	R1=0.0301 wR2=0.0700
Largest diff. peak and hole/e Å^{-3}	0.198/-0.361	0.137/-0.591	0.420/-0.399

2.4 Theoretical calculations

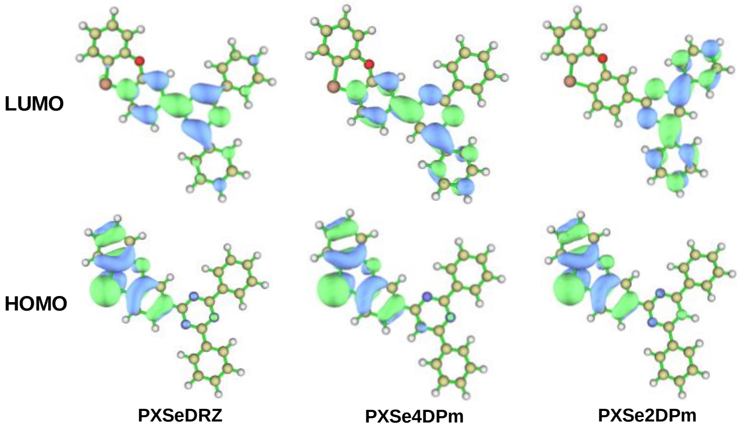

Figure S9. Theoretical HOMO and LUMO distributions of the investigated compounds.

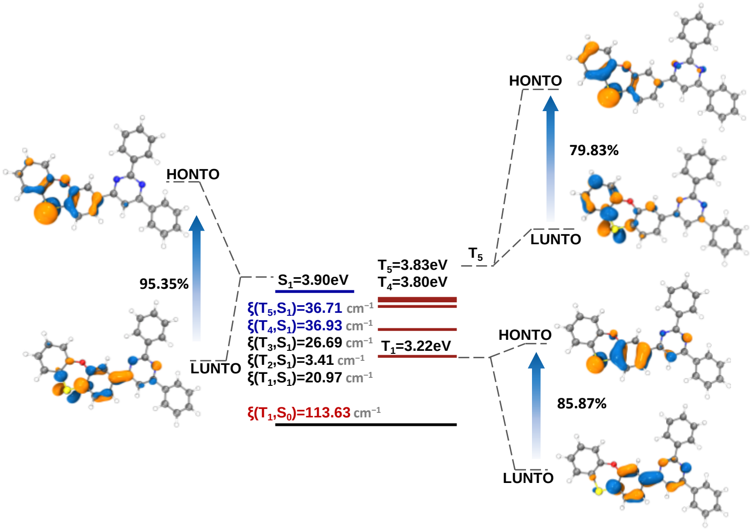

Figure S10. Calculated energy levels, SOC matrix elements, and NTOs of PXSe4DPm.

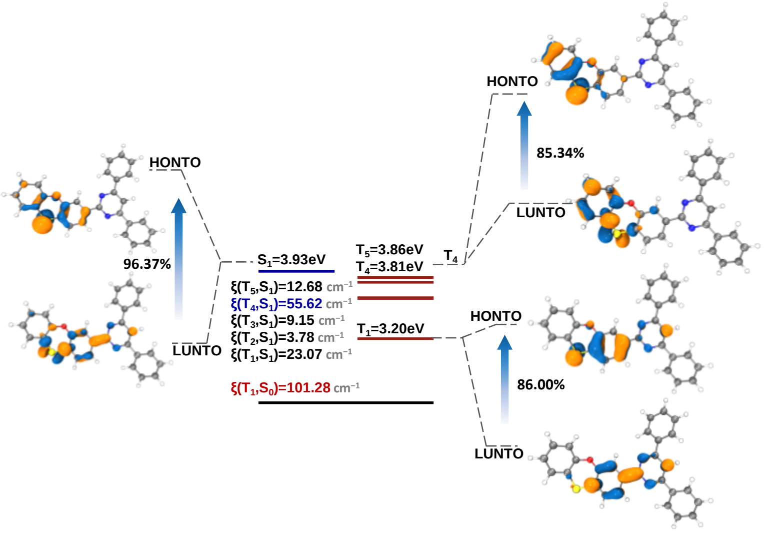

Figure S11. Calculated energy levels, SOC matrix elements, and NTOs of PXSe2DPm.

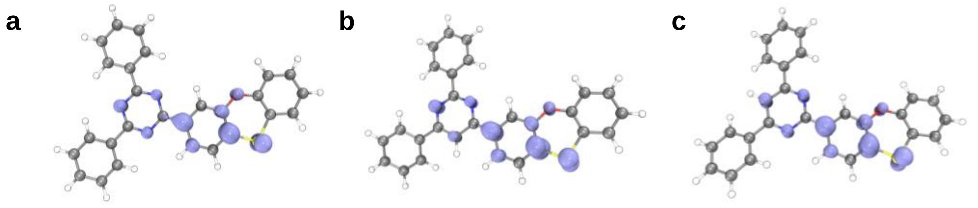

Figure S12. Triplet spin density distributions of the investigated compounds **a** PXSeDRZ, **b** PXSe4DPm and **c** PXSe2DPm.

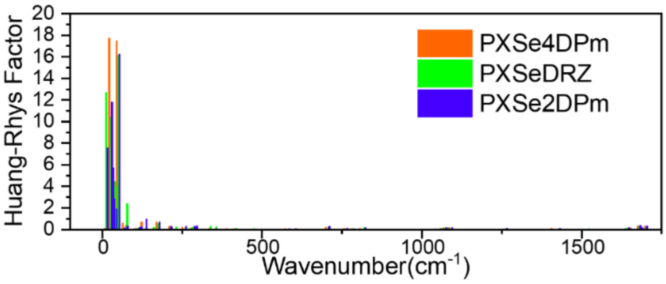

Figure S13. Huang-Rhys factors of different normal vibration modes of PXSeDRZ, PXSe4DPm and PXSe2DPm.

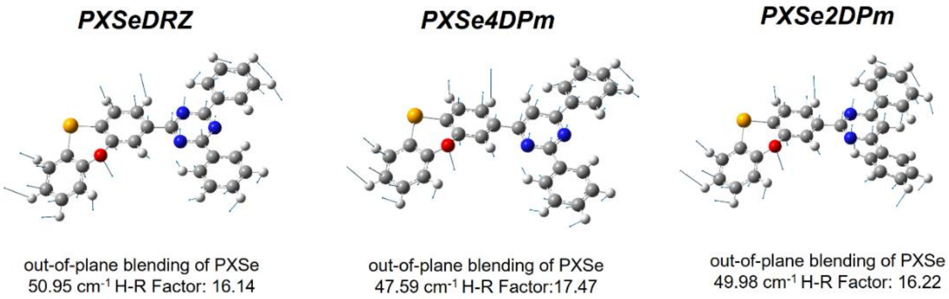

Figure S14. Representative normal vibration modes and corresponding Huang-Rhys (H-R) factors of the investigated molecules.

2.5 Device characterizations

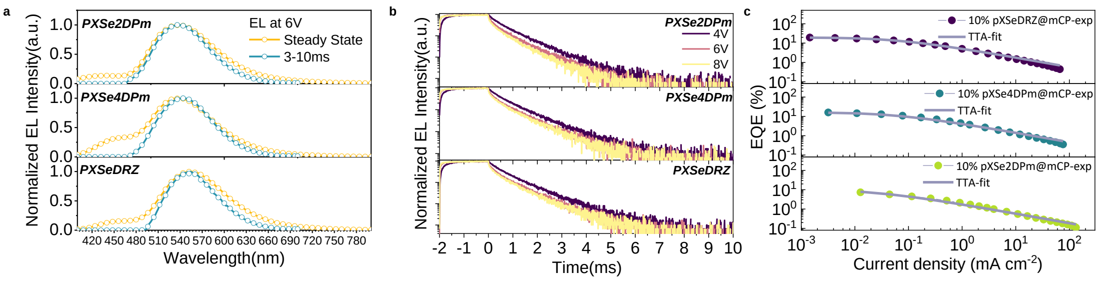

Figure S15. a Steady-state and delayed electroluminescent spectra of the OLEDs detected at 6 V; **b** Transient EL decay spectra of the OLEDs detected at different voltages; **c** External quantum efficiency-current density curves (EQE-J) of OLEDs and the simulated EQE fitted by the TTA model with the architecture of ITO/MoO_3 (3 nm)/TAPC (30 nm)/mCP (10 nm)/10 wt.% emitter: mCP (20 nm)/TmPyPB (80 nm)/LiF (1 nm)/Al.

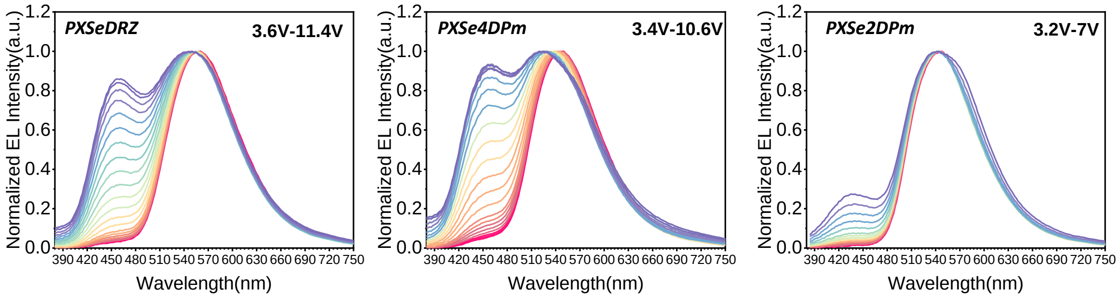

Figure S16. EL spectra at different voltages of the OLEDs with the architecture of ITO/MoO_3 (3 nm)/TAPC (30 nm)/mCP (10 nm)/10 wt.% emitter: mCP (20 nm)/TmPyPB (80 nm)/LiF (1 nm)/Al.

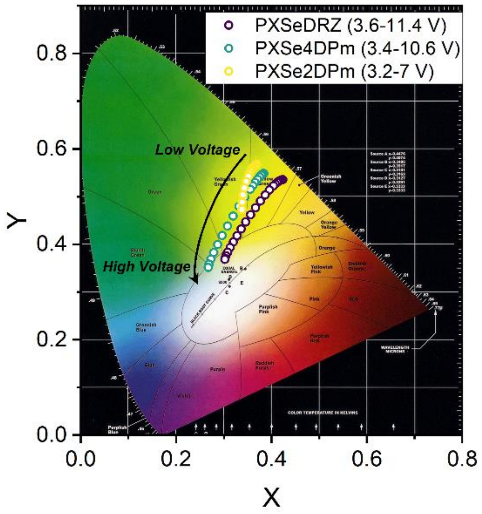

Figure S17. Commission Internationale de L'Eclairage (CIE) coordinates of the OLED devices under different voltages with the architecture of ITO/MoO_3 (3 nm)/TAPC (30 nm)/mCP (10 nm)/10 wt.% emitter: mCP (20 nm)/TmPyPB (80 nm)/LiF (1 nm)/Al.

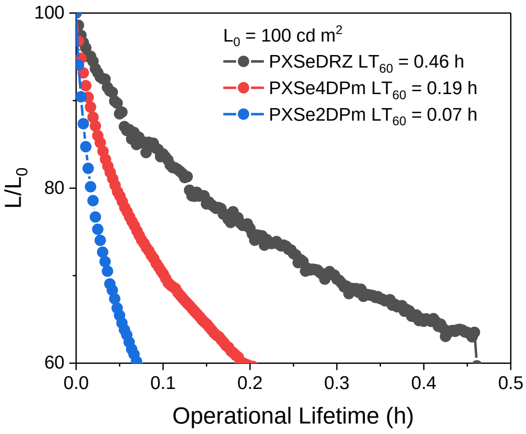

Figure S18. The operational lifetimes of the devices with an initial luminance (L_0) of 100 cd m^2 with the architecture of ITO/MoO_3 (3 nm)/TAPC (30 nm)/mCP (10 nm)/10 wt.% emitter: mCP (20 nm)/TmPyPB (80 nm)/LiF (1 nm)/Al.

Table S5. Summary of the detailed device performances in which V_{on}, L_{max}, CE_{max}, PE_{max}, EQE_{max}, and CIE denotes turn-on voltage, maximum luminance, maximum current efficiency, maximum power efficiency, maximum EQE, and CIE coordinates, respectively.

	V_{on}[a] [V]	L_{max} [cd m^{-2}]	CE_{max} [cd A^{-1}]	PE_{max} [lm W^{-1}]	EQE_{max}	CIE(x,y) [b]
PXSeDRZ	3.6	687.4	58.7	53.3	19.5%	(0.42,0.53)
PXSe4DPm	3.4	598.4	50.6	46.7	16.0%	(0.38,0.55)
PXSe2DPm	3.2	342.1	23.6	23.2	7.3%	(0.37,0.57)

[a] Turn on voltage at the luminance of 1 cd m^{-2}; [b] CIE coordinates at the luminance of 1 cd m^{-2}.

Table S6. Summary of the detailed device performances of the devices based on PXSeDRZ and PXSe4DPm under high applied voltages.

	V[a] [V]	L [cd m^{-2}]	CE [cd A^{-1}]	PE [lm W^{-1}]	EQE [%]	CIE (x, y)
PXSeDRZ	10.6	687.4	1.0	0.3	0.5	(0.30, 0.37)
PXSe4DPm	9.0	598.4	0.89	0.3	0.4	(0.27, 0.35)

[a] Applied voltage of the devices.

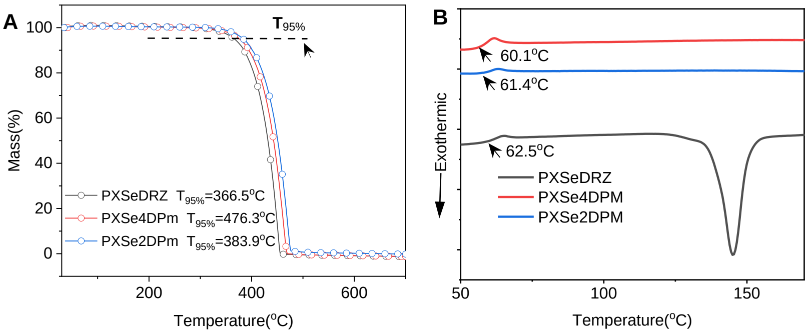

Figure S19. a Thermal properties of the investigated compounds PXSeDRZ, PXSe4DPm, and PXSe2DPm. **b** Corresponding thermal decomposition temperature with 95% mass loss ($T_{95\%}$) and glassy transition temperature (T_g) were marked by black arrows as shown.

2.6 Energy levels

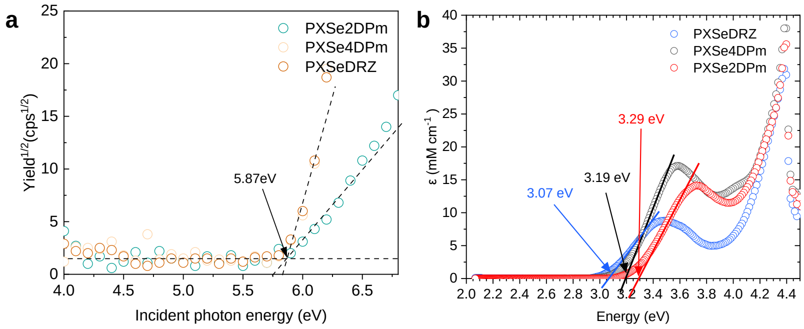

Figure S20. a Photoelectron yield spectra (AC-3) of vacuum evaporation prepared neat films of the investigated compounds. **b** UV-Vis absorption spectra in toluene solvent.

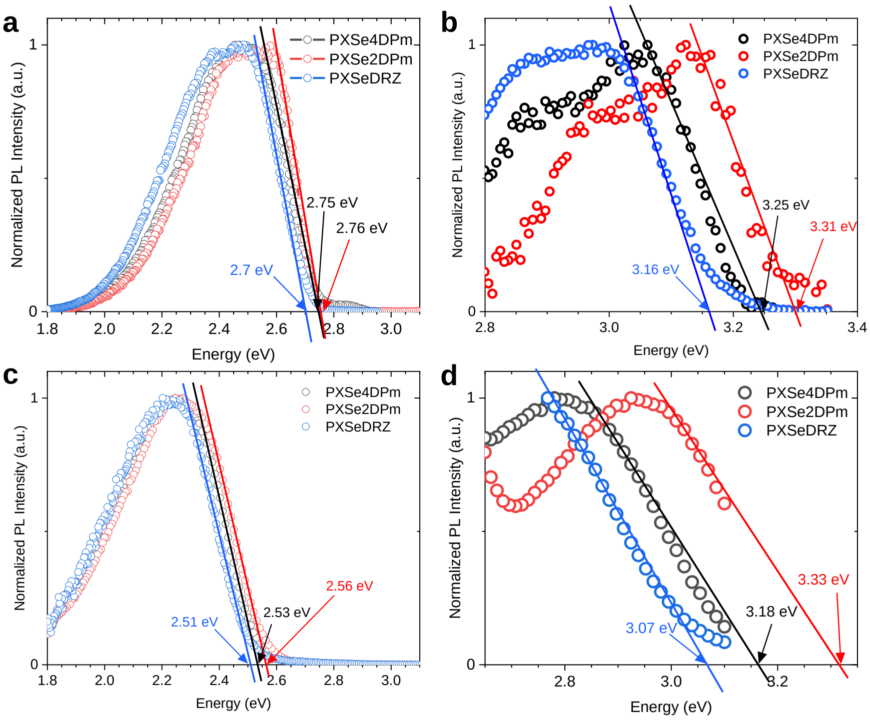

Figure S21. a Phosphorescence spectra, **b** Steady-state spectra of the investigated compounds in toluene solvent at 77 K; **c** Phosphorescence spectra, **d** Steady-state spectra of the investigated compounds in doped mCP films at 298 K in vacuum with the calculated excited state energies obtained from the onsets of the emissions.

Table S7. Comparison of the photophysical parameters of the investigated compounds in toluene solvent and 10% doped mCP films.

	S_1 [eV]		T_1 [eV]		ΔE_{ST} [eV]	
	Solution	Doped film	Solution	Doped film	Solution	Doped film
PXSeDRZ	3.16	3.07	2.70	2.51	0.46	0.56
PXSe4DPm	3.25	3.18	2.75	2.53	0.50	0.65
PXSe2DPm	3.31	3.33	2.76	2.56	0.55	0.77

2.7 Identification of the EL emission in OLED devices

The electroluminescent spectra of the corresponding devices consist of fluorescence at around 450 nm and phosphorescence at around 550 nm, corresponding well with their photoluminescent spectra in 10% doped mCP films. Due to the direct 75% triplet exciton generation under electroluminescence, the ratio of phosphorescence at low driving voltage is higher than the ratio under photoluminescence. However, severe triplet exciton quenching is occurred under high driving voltage, and the ratio of fluorescence is enhanced while the phosphorescence is quenched. To identify the origin of the EL emission in OLED devices and further exclude the possibility of interfacial exciplex emission of mCP and TmPyPB, photophysical investigations under photoluminescence and electroluminescence have been carried out. Firstly, no obvious exciplex emission can be observed in the mCP:TmPyPB (1:1) mixed film. The emission is merely a simple addition of the emissions of the mCP and TmPyPB neat films, and no obvious emission of intermolecular CT transition is observed in the long wavelength region (Figure S22). OLED device with the architecture of ITO/MoO_3 (3 nm)/TAPC (30 nm)/mCP (30 nm) /TmPyPB (80 nm)/LiF (1 nm)/Al was fabricated without emitters. Similar emission is observed under electroluminescence without obvious exciplex emission with interfacial CT character, indicating there is no interfacial exciplex emission (Figure S23). Further, non-doped OLED devices with the architecture of ITO/MoO_3 (3 nm)/TAPC (30 nm)/mCP (10 nm)/ emitter/TmPyPB (80 nm)/LiF (1 nm)/Al were fabricated to investigate the emission of emitters under electroluminescence, and OLED devices with the architecture of ITO/MoO_3 (3 nm)/TAPC (30 nm)/mCP (10 nm)/10 wt.% emitter: mCP (20 nm)/DPEPO (10 nm)/TmPyPB (80 nm)/LiF (1 nm)/Al with a layer of DPEPO inserted between the emission layer and TmPyPB were also fabricated to investigate the

emission in 10% doped mCP film (Figure S24). Similar dual emissions of fluorescence and phosphorescence were observed in the non-doped devices and the devices with DPEPO, confirming that the dual emissions are attributed to fluorescence and phosphorescence of the emitters rather than interfacial exciplex emission or TADF emission. According to the experimental results, the emissions at around 450 nm are originated from fluorescence of the emitters and the emissions at around 550 nm are originated from phosphorescence of the emitters in the OLED devices.

To exclude the possibility to be TAPC excimer emission in OLED devices, transient electroluminescence (EL) spectra of the TAPC excimer and device performance of the OLED device without RTP emitters have been newly added (Figure S25). Combined with the steady state, delayed spectra and transient EL decay spectra, the excimer emission in OLED devices can be rationally excluded. The PL and EL spectra of PXSeDRZ exhibited similar emission at a peak of 560 nm and the TAPC excimer exhibited totally different emission at a peak of 580 nm (Figure S25a). Since the emission of the TAPC excimer is fluorescence, its corresponding EL emission lifetime is very short (in nanosecond timescale) (Figure S25d). And the transient EL decay spectra of the OLED devices (Figure S25e) showed long lifetime of millisecond timescale, excluding the fluorescence emission character. Also, with a time delay of 3-10 ms, strong EL spectra can still be recorded, and the shape and peak of the delayed EL spectra correspond well with the phosphorescence in steady state EL spectra (Figure S25f). Moreover, compared with the OLED devices without RTP emitters exhibited the emission from the TAPC excimer, the OLED devices based on the investigated RTP compounds behave completely different device characteristics and much higher external quantum efficiencies (Figure S25b and S25c), indicating that the long-lived electroluminescence in the OLED devices should be electro-phosphorescence rather than the fluorescence of the TAPC excimer.

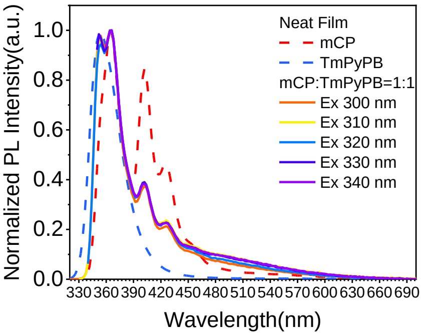

Figure S22. PL spectra of mCP, TmPyPB neat film and mCP:TmPyPB (1:1) mixed film under different excitation wavelengths.

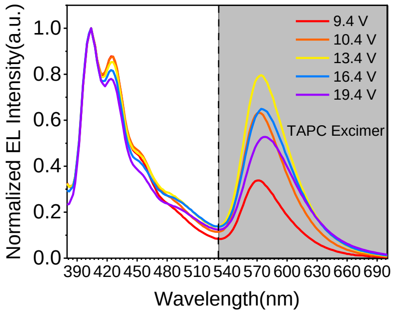

Figure S23. EL spectra of the OLED device with the architecture of ITO/MoO_3 (3 nm)/TAPC (30 nm)/mCP (30 nm) /TmPyPB (80 nm)/LiF (1 nm)/Al at various voltages.

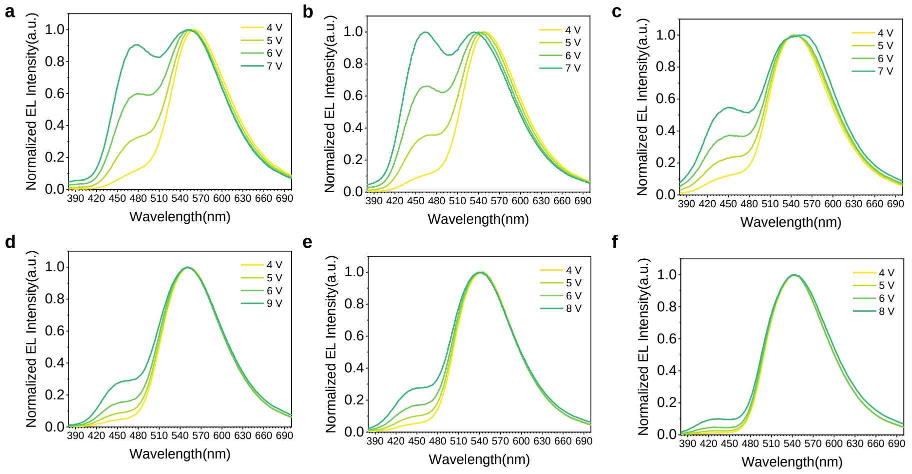

Figure S24. EL spectra of the OLED devices of **a** PXSeDRZ, **b** PXSe4DPm, **c** PXSe2DPm with the architecture of ITO/MoO_3 (3 nm)/TAPC (30 nm)/mCP (10 nm)/10 wt.% emitter: mCP (20 nm)/DPEPO (10 nm)/TmPyPB (80 nm)/LiF (1 nm)/Al at various voltages, and EL spectra of the OLED devices of **d** PXSeDRZ, **e** PXSe4DPm, **f** PXSe2DPm with the architecture of ITO/MoO_3 (3 nm)/TAPC (30 nm)/mCP (10 nm)/ emitter/TmPyPB (80 nm)/LiF (1 nm)/Al at various voltages.

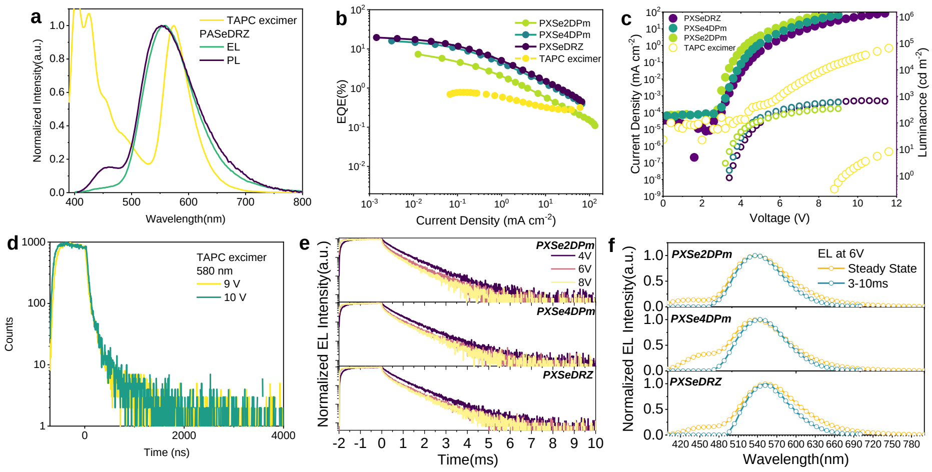

Figure S25. a Steady state PL spectra of PXSeDRZ in 10% doped mCP film and steady state EL spectra of PXSeDRZ and TAPC excimer in OLED devices; **b** External quantum efficiency-current density curves (EQE-J) of the OLED devices; **c** Current density-voltage-luminance (J-V-L) curves of the OLED devices; **d** Transient EL decay spectra of the OLED devices detected at different voltages in Device B of the TAPC excimer; **e** Transient EL decay spectra of the OLED devices detected at different voltages in Device A; **f** Steady-state and delayed electroluminescent spectra of the OLED devices detected at 6 V in Device A. Device A: ITO/MoO_3 (3 nm)/TAPC (30 nm)/mCP (10 nm)/10 wt.% emitter: mCP (20 nm)/TmPyPB (80 nm)/LiF (1 nm)/Al, Device B: ITO/MoO_3 (3 nm)/TAPC (30 nm)/mCP (10 nm)/ mCP (20 nm)/TmPyPB (80 nm)/LiF (1 nm)/Al.

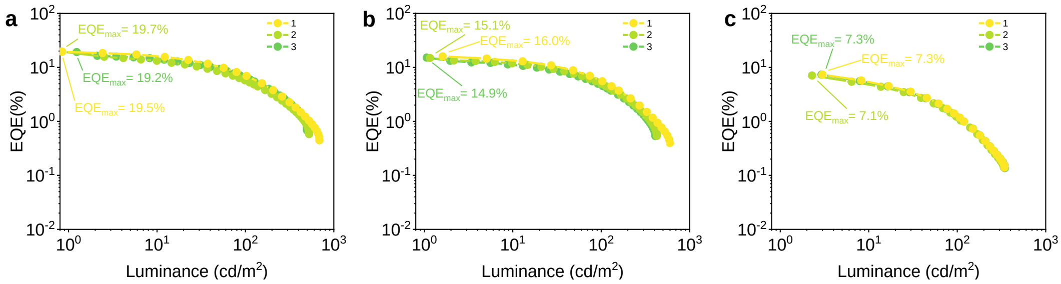

Figure S26. The repeatability of device performances of the present devices has been confirmed by three different samples. External quantum efficiency-luminance curves (EQE-L) of the OLED devices of **a** PXSeDRZ, **b** PXSe4DPm, **c** PXSe2DPm with the architecture of ITO/MoO_3 (3 nm)/TAPC (30 nm)/mCP (10 nm)/10 wt.% emitter: mCP (20 nm)/TmPyPB (80 nm)/LiF (1 nm)/Al in three different samples.

2.8 Further identification of the origin of the emissions

The origin of the emissions in the doped films can be further evidenced by: 1) The transient phosphorescence decay spectra recorded in nanosecond scale without prompt component indicate the pure phosphorescence nature (Figure S27a, S27b and S27c). 2) The individual PL spectra of the phenoxaselenine and s-Triazine moieties measured in toluene (Figure S27d) show that the phenoxaselenine moiety shows localized (LE) fluorescence emission and phosphorescence emissions with peaks of 384 and 431 nm, and the s-Triazine moiety shows fluorescence and phosphorescence emissions with peaks of 408 and 440 nm, which is completely different from the luminescence of the investigated compounds. Also, the dual emissions of the investigated compounds exhibit obvious charge-transfer (CT) character with broad emission spectra and significant solvation effect (Figure S27e) which are totally different from the LE characters of moieties. Overall, the possibility of luminescence coming from different moieties can also be ruled out with certainty.

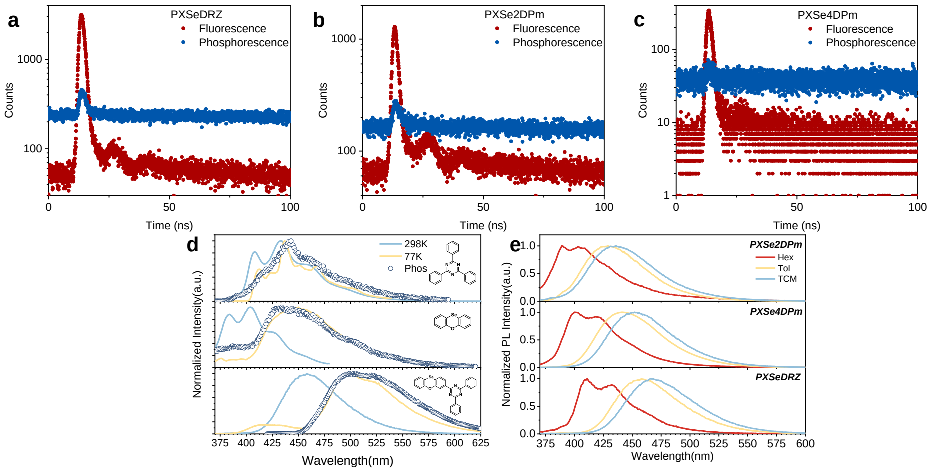

Figure S27. Transient fluorescence and phosphorescence decay spectra in nanosecond scale of **a** PXSeDRZ, **b** PXSe2DPm and **c** PXSe4DPm; **d** Steady-state PL (at 298 K and 77 K) and phosphorescence (at 77 K with a delayed time of 5 ms) spectra of PXSeDRZ, phenoxaselenine and s-Triazine in toluene (10^{-5} M); **e** PL spectra of PXSe2DPm, PXSe4DPm, and PXSeDRZ in n-hexane, toluene, and trichloromethane (10^{-5} M).

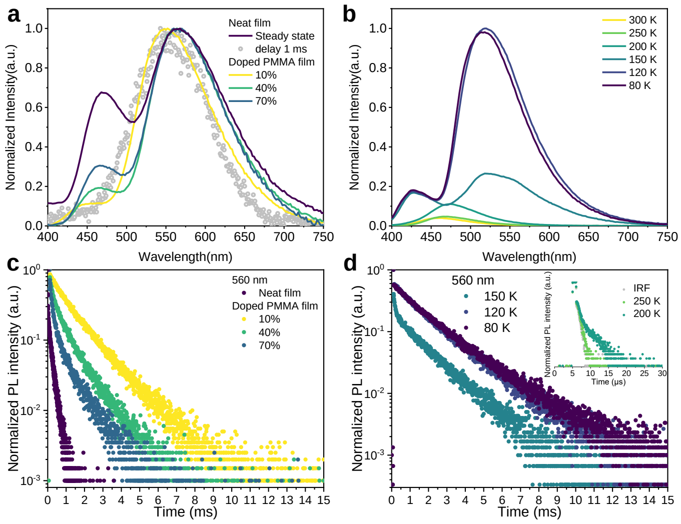

Figure S28. a Steady state PL spectra of the PXSeDRZ neat film and PXSeDRZ doped PMMA films with concentrations of 10%, 40% as well as 70% and PL spectrum of the PXSeDRZ neat film with a delayed time of 1 ms; **b** Temperature-dependent PL spectra of PXSeDRZ in mesitylene (10^{-5} M); **c** Transient phosphorescence decay spectra of the PXSeDRZ neat film and PXSeDRZ doped PMMA films with concentrations of 10%, 40% as well as 70%; **d** Temperature-dependent transient phosphorescence decay spectra of PXSeDRZ in Mesitylene (10^{-5} M).

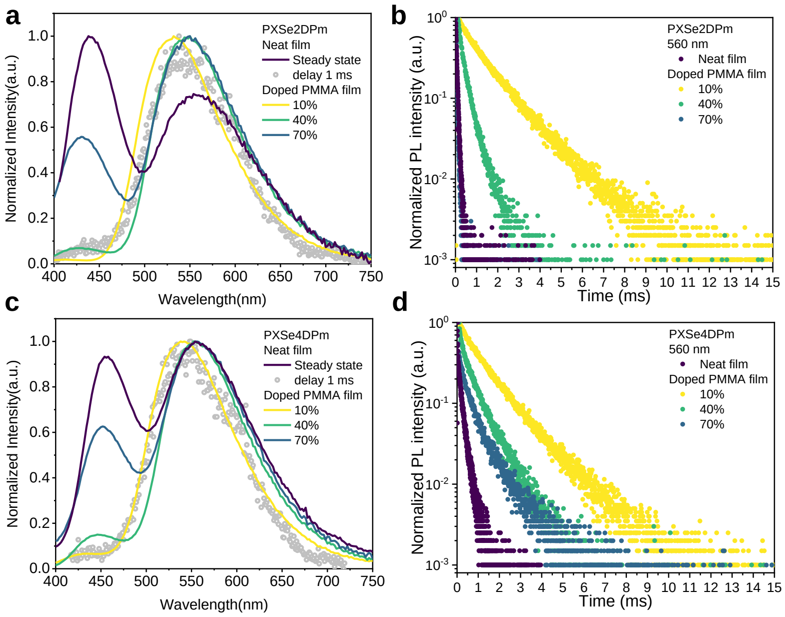

Figure S29. a Steady state PL spectra of the PXSe2DPm neat film and PXSe2DPm doped PMMA films with concentrations of 10%, 40% as well as 70% and PL spectrum of the PXSe2DPm neat film with a delayed time of 1 ms; **b** Transient phosphorescence decay spectra of the PXSe2DPm neat film and PXSe2DPm doped PMMA films with concentrations of 10%, 40% as well as 70%; **c** Steady state PL spectra of the PXSe4DPm neat film and PXSe4DPm doped PMMA films with concentrations of 10%, 40% as well as 70% and PL spectrum of the PXSe4DPm neat film with a delayed time of 1 ms; **d** Transient phosphorescence decay spectra of the PXSe4DPm neat film and PXSe4DPm doped PMMA films with concentrations of 10%, 40% as well as 70%.

3 Figures of the chemical structure characterizations

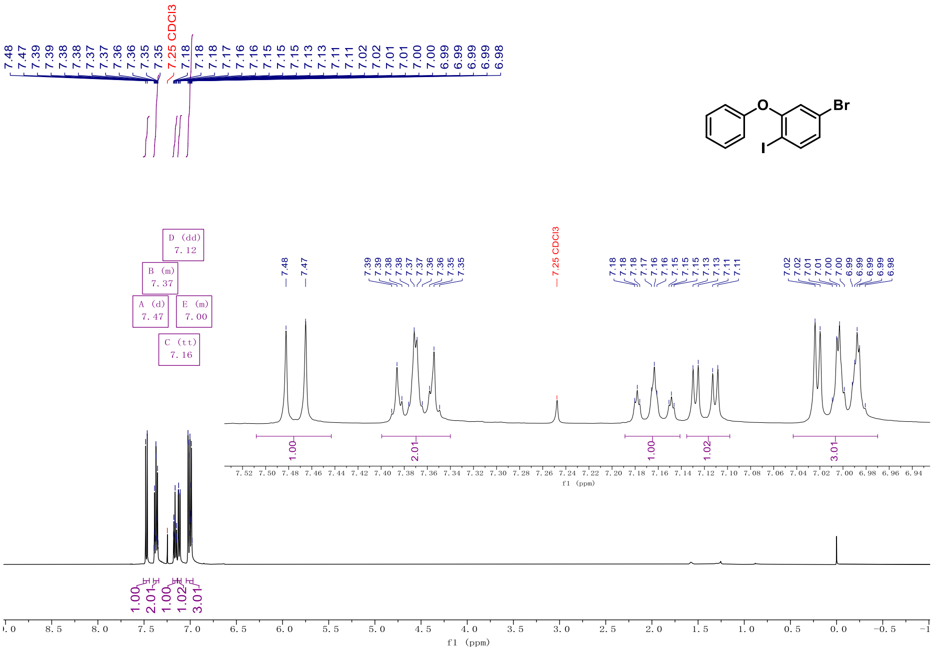

Figure S30. ^{1}H NMR spectrum of compound 4-bromo-1-iodo-2-phenoxybenzene (1) measured in deuterated $CDCl_3$.

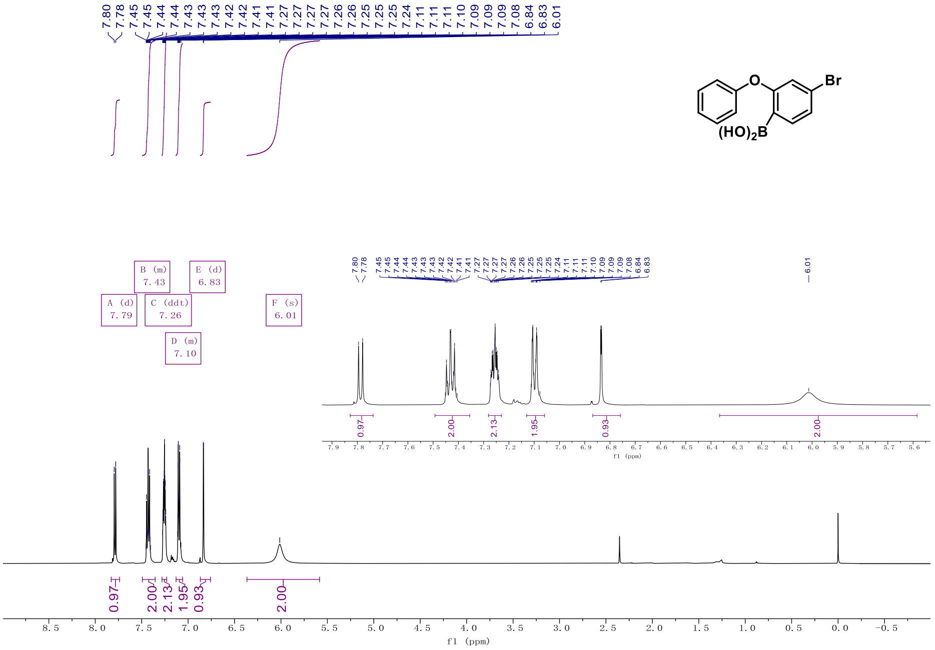

Figure S31. ^{1}H NMR spectrum of compound (4-bromo-2-phenoxyphenyl)boronic acid (2) measured in deuterated $CDCl_3$.

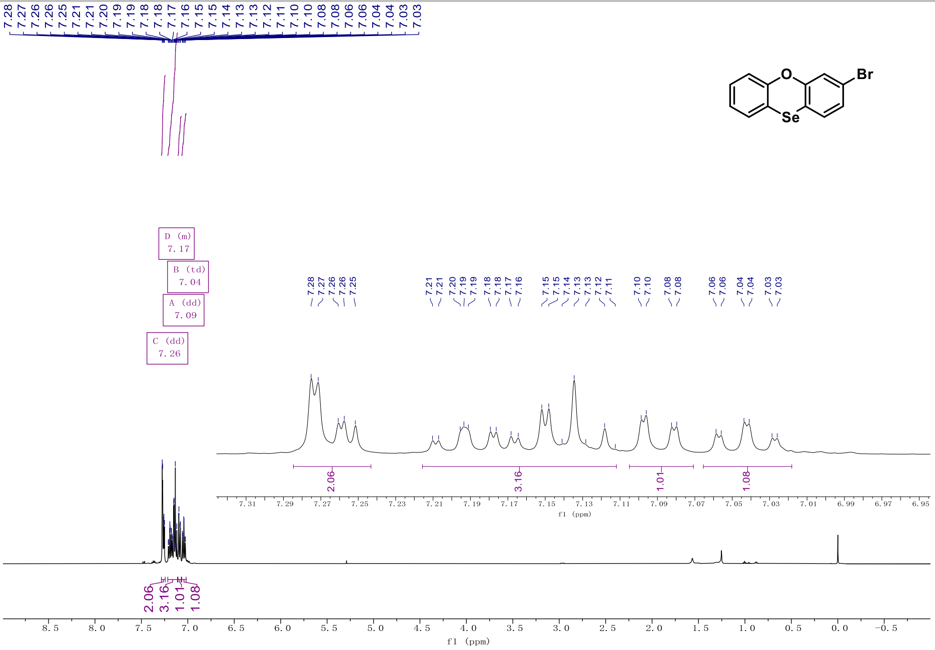

Figure S32. ^{1}H NMR spectrum of compound 3-bromophenoxaselenine (3) measured in deuterated $CDCl_3$.

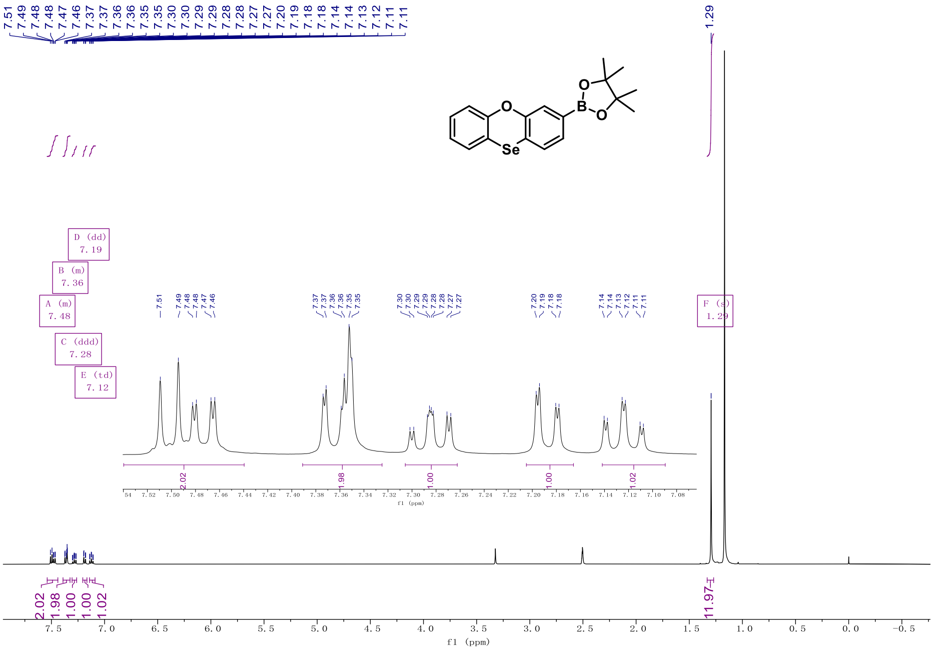

Figure S33. ^{1}H NMR spectrum of compound 4,4,5,5-tetramethyl-2-(phenoxaselenin-3-yl)-1,3,2-dioxaborolane (4) measured in deuterated DMSO-d6.

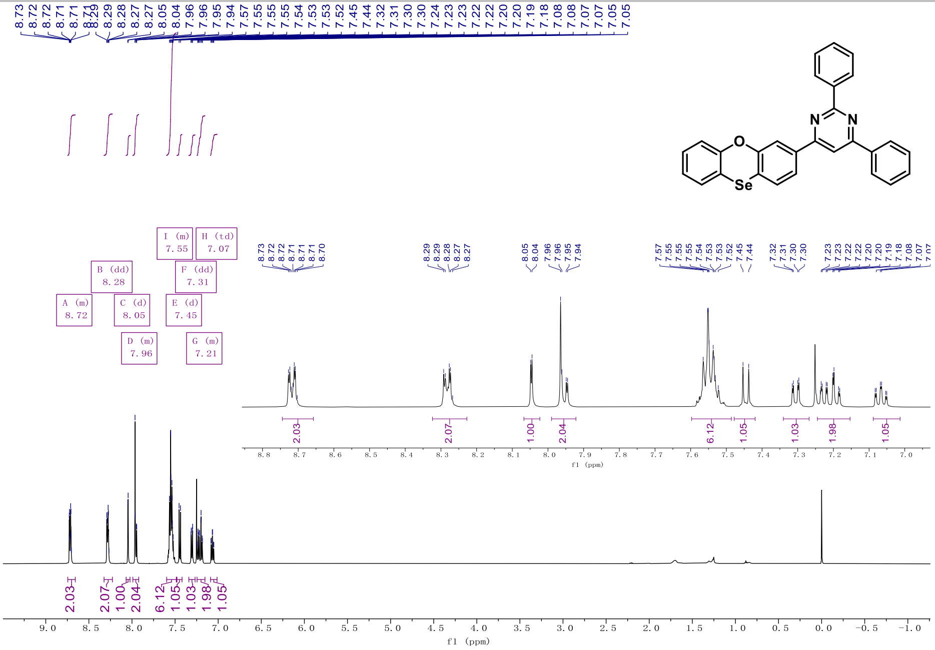

Figure S34. ^{1}H NMR spectrum of compound 4-(phenoxaselenin-3-yl)-2,6-diphenylpyrimidine (PXSe4DPm) measured in deuterated $CDCl_3$.

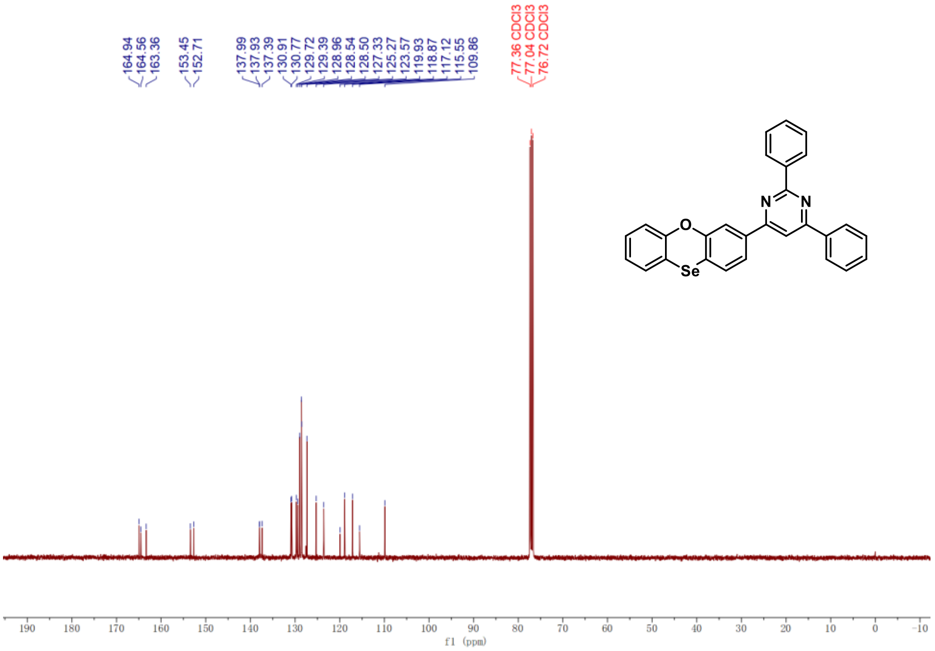

Figure S35. ^{13}C NMR spectrum of compound 4-(phenoxaselenin-3-yl)-2,6-diphenylpyrimidine (PXSe4DPm) measured in deuterated $CDCl_3$.

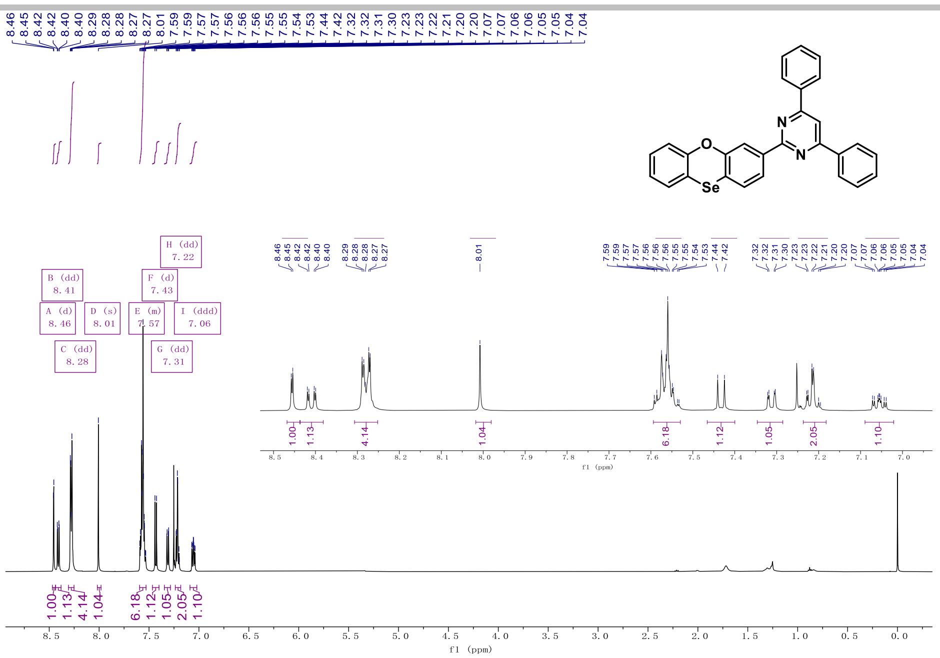

Figure S36. ^{1}H NMR spectrum of compound 2-(phenoxaselenin-3-yl)-4,6-diphenylpyrimidine (PXSe2DPm) measured in deuterated $CDCl_3$.

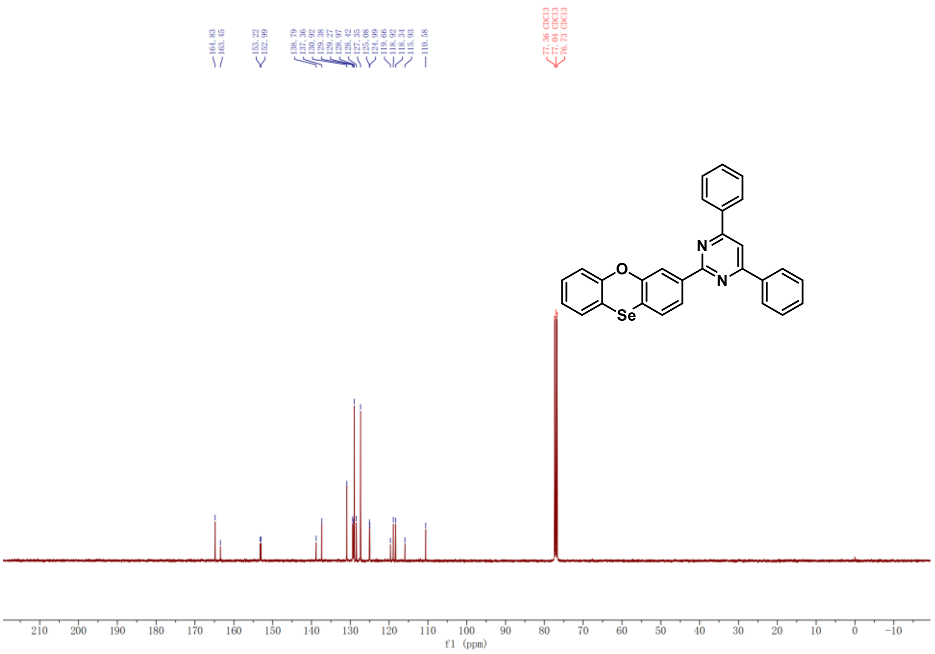

Figure S37. ^{13}C NMR spectrum of compound 2-(phenoxaselenin-3-yl)-4,6-diphenylpyrimidine (PXSe2DPm) measured in deuterated $CDCl_3$.

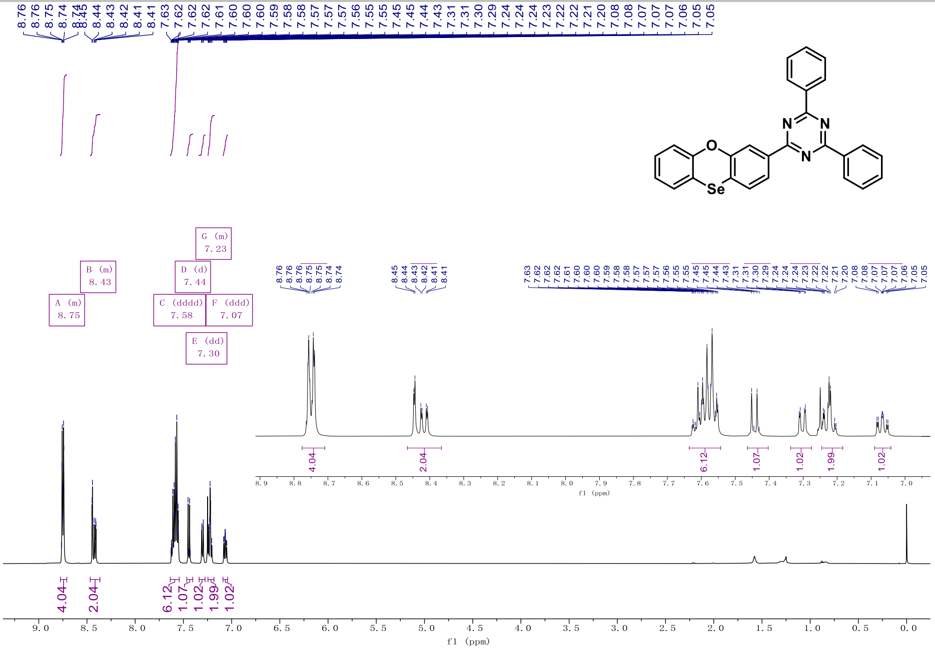

Figure S38. ^{1}H NMR spectrum of compound 2-(phenoxaselenin-3-yl)-4,6-diphenyl-1,3,5-triazine (PXSeDRZ) measured in deuterated $CDCl_3$.

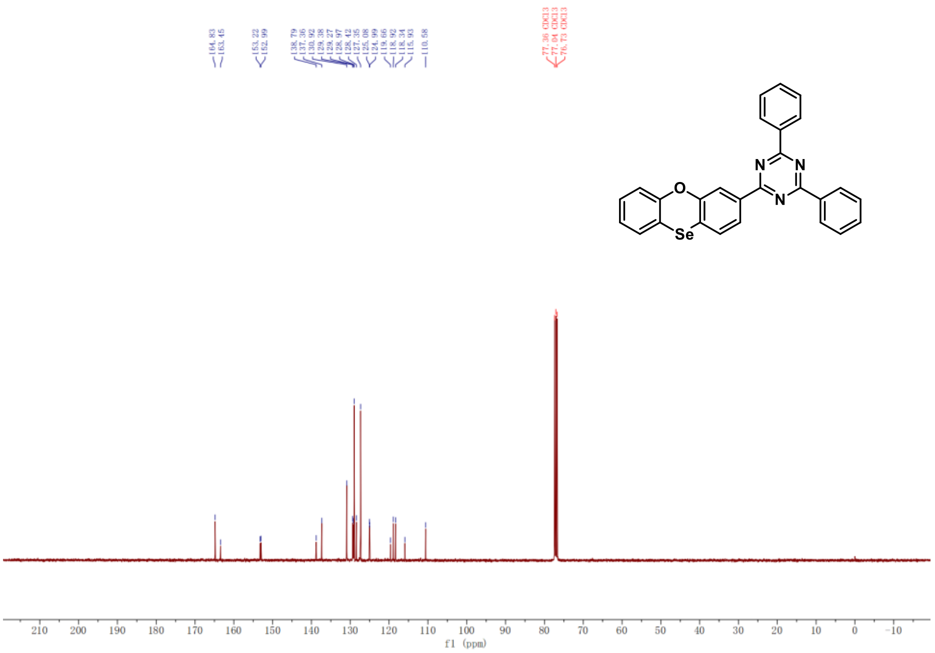

Figure S39. ^{13}C NMR spectrum of compound 2-(phenoxaselenin-3-yl)-4,6-diphenyl-1,3,5-triazine (PXSeDRZ) measured in deuterated $CDCl_3$.

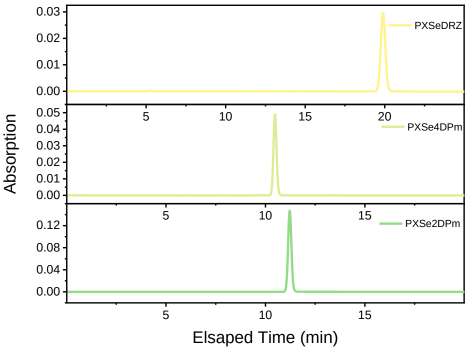

Figure S40. HPLC measurements of the investigated compounds.

4 References

[1] X. Zhang, X. B. Huang, Y. B. Zhou, M. C. Liu, H. Y. Wu, *Chem. Eur. J.* **2021**, *27*, 944-948.

5 Author Contributions

Z. Chen and M. Li conducted the project and prepared the manuscript. Z. Chen, M. Li, Y. Jiao contributed to the synthesis and characterization. W. Qiu, W. Xie contributed to the optical measurement. Q. Gu, X. Peng and D. Liu fabricated and characterized the devices. W. Qiu and K. Liu contributed to the analyses of results. J. Zhou contributed to the single crystal X-ray analyses. S.-J. Su supervised the project and revised the manuscript.

6 Molecular coordinates

S0 geometry of PXSeDRZ

C	-5.32013	-0.1233	0.18245
C	-4.54893	1.03428	0.04778
C	-6.54957	-0.05846	0.83974
C	-7.01978	1.15774	1.3314
C	-6.24925	2.31095	1.18531
C	-5.00916	2.24765	0.55531
O	-3.34366	1.04425	-0.60356
C	-2.45586	0.01595	-0.42644
C	-2.85715	-1.32381	-0.38062
Se	-4.69962	-1.75161	-0.59385
C	-1.11024	0.34911	-0.32881
C	-0.14674	-0.65566	-0.20242
C	-0.54486	-1.99627	-0.15099
C	-1.89378	-2.32385	-0.22892
C	1.29089	-0.29414	-0.11354
N	2.17796	-1.28126	0.00849
C	3.45747	-0.91256	0.08508
N	3.86795	0.3554	0.04654
C	2.91515	1.27981	-0.07674
N	1.61433	0.99769	-0.16021
C	3.3252	2.7068	-0.12311
C	4.48517	-1.97675	0.2212
C	2.35945	3.71181	-0.25597
C	2.74502	5.04838	-0.29875
C	4.09494	5.39013	-0.20928
C	5.06032	4.39129	-0.07694
C	4.67879	3.05354	-0.03396
C	5.84117	-1.63799	0.30388
C	6.80307	-2.6358	0.43092
C	6.4186	-3.97636	0.47622
C	5.06815	-4.31794	0.39419
C	4.10328	-3.32296	0.26702
H	-7.14194	-0.96732	0.9602
H	-7.98702	1.20082	1.83278
H	-6.60991	3.26505	1.57118
H	-4.37814	3.12812	0.43293
H	-0.81656	1.39712	-0.36494
H	0.21323	-2.77073	-0.044
H	-2.20816	-3.36752	-0.17613
H	1.3101	3.42662	-0.32481
H	1.98952	5.82831	-0.40269
H	4.39571	6.43854	-0.24292
H	6.1159	4.65734	-0.00705
H	5.41864	2.26028	0.06889
H	6.1229	-0.58613	0.2667
H	7.85847	-2.36759	0.49488
H	7.17389	-4.75763	0.57575
H	4.7664	-5.36561	0.42944
H	3.0444	-3.57128	0.2014

S0 geometry of PXSe4DPm

C	-5.3619	-0.17207	0.08774
C	-4.57126	0.9237	-0.26887
C	-6.60709	0.04149	0.68065
C	-7.07303	1.33841	0.88752
C	-6.28302	2.42744	0.52041
C	-5.02778	2.22113	-0.04577
O	-3.34806	0.78515	-0.87123
C	-2.47995	-0.18723	-0.4521
C	-2.90243	-1.48189	-0.13186
Se	-4.74345	-1.93328	-0.30608
C	-1.1332	0.14981	-0.38711
C	-0.17882	-0.80704	-0.02746
C	-0.60465	-2.09914	0.30514
C	-1.9558	-2.42766	0.26437

C	1.2569	-0.41749	0.00763
C	2.28402	-1.36601	0.02013
C	3.59949	-0.89123	0.05802
N	3.85015	0.41832	0.05818
C	2.81416	1.25708	0.02952
N	1.53374	0.88684	0.0089
C	3.10988	2.71686	0.0261
C	4.77031	-1.80981	0.07722
C	2.06635	3.64935	-0.01581
C	2.34519	5.01324	-0.02134
C	3.66677	5.45851	0.01684
C	4.70989	4.5332	0.06202
C	4.43446	3.16863	0.0671
C	6.02932	-1.32677	-0.30212
C	7.1369	-2.16941	-0.30254
C	7.00436	-3.50261	0.08649
C	5.75748	-3.9886	0.47787
C	4.64708	-3.14803	0.47195
H	-7.21483	-0.81589	0.9756
H	-8.05229	1.49555	1.34044
H	-6.64041	3.4448	0.68313
H	-4.38142	3.04997	-0.33494
H	-0.82562	1.16477	-0.63457
H	0.11142	-2.85464	0.62787
H	-2.28287	-3.4304	0.54419
H	2.07213	-2.43045	-0.044
H	1.03939	3.28628	-0.04153
H	1.52664	5.73373	-0.05461
H	3.884	6.52789	0.01304
H	5.74459	4.87758	0.09481
H	5.2378	2.43358	0.10645
H	6.11735	-0.28204	-0.59953
H	8.11065	-1.78392	-0.60805
H	7.87364	-4.16192	0.0891
H	5.64933	-5.02639	0.79599
H	3.68333	-3.537	0.80318

S0 geometry of PXSe2DPm

C	-5.26534	-0.19333	0.21168
C	-4.51185	0.97612	0.0753
C	-6.49142	-0.14817	0.87687
C	-6.97669	1.05948	1.37502
C	-6.22409	2.22432	1.22752
C	-4.98717	2.18097	0.58964
O	-3.31181	1.00586	-0.58372
C	-2.40596	-0.00908	-0.41035
C	-2.78718	-1.35383	-0.36383
Se	-4.62443	-1.80871	-0.57453
C	-1.06565	0.34598	-0.31381
C	-0.08422	-0.64134	-0.18868
C	-0.4639	-1.98732	-0.13277
C	-1.80763	-2.33705	-0.20968
C	1.35137	-0.25629	-0.10302
N	2.24402	-1.23821	0.01811
C	3.5339	-0.90627	0.09317
C	3.92932	0.43418	0.03709
C	2.9251	1.40141	-0.07488
N	1.64225	1.04327	-0.14778
C	3.23166	2.8561	-0.1395
C	4.51763	-2.01617	0.21496
C	2.27464	3.73828	-0.65742
C	2.54283	5.1015	-0.73749
C	3.76563	5.60343	-0.2912
C	4.71906	4.73421	0.23756
C	4.45452	3.36896	0.31166
C	4.13848	-3.31452	-0.14967
C	5.0444	-4.36661	-0.05436
C	6.33758	-4.13842	0.41676
C	6.71966	-2.85157	0.79386
C	5.81607	-1.79675	0.69225
H	-7.06927	-1.06621	0.99846
H	-7.94129	1.08679	1.88261
H	-6.59616	3.17189	1.61871
H	-4.36966	3.07088	0.46643
H	-0.78962	1.3989	-0.34594
H	0.3057	-2.74942	-0.01927
H	-2.10473	-3.38566	-0.15282
H	4.98081	0.71112	0.03272
H	1.32264	3.33188	-0.99825
H	1.79286	5.77749	-1.15061
H	3.97408	6.67267	-0.35125
H	5.67204	5.12151	0.6005
H	5.20129	2.70499	0.74897
H	3.12387	-3.47767	-0.51235
H	4.74005	-5.37184	-0.34931
H	7.04647	-4.96411	0.49436
H	7.725	-2.66843	1.17552
H	6.12267	-0.80025	1.01286

S1 geometry of PXSeDRZ

C	-5.39012	-0.18345	-0.02068
C	-4.62066	0.99672	-0.21669
C	-6.8016	-0.09522	-0.05631
C	-7.42617	1.10674	-0.29624
C	-6.654	2.28016	-0.48151
C	-5.27985	2.22521	-0.43448
O	-3.29004	1.06377	-0.15704
C	-2.42342	0.02316	-0.07761
C	-2.84572	-1.34481	-0.11412
Se	-4.58582	-1.79103	0.43228
C	-1.07955	0.36186	-0.04199
C	-0.09743	-0.63483	-0.04724
C	-0.51017	-2.01098	-0.109
C	-1.83892	-2.34537	-0.12301
C	1.30962	-0.27584	-0.02406
N	2.22053	-1.26747	-0.06038
C	3.49264	-0.8914	-0.04364
N	3.90747	0.37894	0.00472
C	2.93687	1.30262	0.03612
N	1.64134	1.02948	0.02592
C	3.34846	2.73264	0.08797
C	4.53306	-1.9562	-0.08074
C	2.38241	3.74478	0.13698
C	2.76923	5.08073	0.18535
C	4.12362	5.41815	0.18485
C	5.0897	4.41315	0.1363
C	4.70551	3.0754	0.08842
C	5.89028	-1.61436	-0.07002
C	6.86318	-2.60996	-0.10485
C	6.48927	-3.95316	-0.15028
C	5.13705	-4.29847	-0.1609
C	4.16238	-3.30543	-0.12645
H	-7.38919	-1.00217	0.10053
H	-8.51394	1.15501	-0.34192
H	-7.14984	3.23464	-0.65939
H	-4.6593	3.11155	-0.56713
H	-0.79258	1.41303	-0.06091
H	0.26137	-2.77796	-0.15024
H	-2.13726	-3.39668	-0.14199
H	1.33021	3.46166	0.13686
H	2.01145	5.86482	0.22403
H	4.42603	6.46605	0.2226
H	6.14919	4.67375	0.13593
H	5.44501	2.27617	0.05047
H	6.16206	-0.55987	-0.0341
H	7.91955	-2.33716	-0.09651
H	7.25262	-4.73261	-0.17763
H	4.84193	-5.34829	-0.19638
H	3.10173	-3.55502	-0.13427

S1 geometry of PXSe4DPm

C	-5.42658	-0.23829	0.00469

C	-4.64222	0.92111	0.25924
C	-6.83673	-0.13012	0.02301
C	-7.44817	1.07011	0.3024
C	-6.66127	2.22411	0.54175
C	-5.28766	2.15081	0.5117
O	-3.30996	0.97049	0.22281
C	-2.4596	-0.0807	0.11464
C	-2.91011	-1.44148	0.11989
Se	-4.63942	-1.83709	-0.51473
C	-1.10983	0.23341	0.08425
C	-0.13633	-0.77201	0.07354
C	-0.58256	-2.14047	0.11407
C	-1.91712	-2.45387	0.11346
C	1.27354	-0.40116	0.05261
C	2.31111	-1.35481	0.07808
C	3.62418	-0.88849	0.06565
N	3.8942	0.42062	0.01083
C	2.85493	1.26168	-0.01275
N	1.57675	0.91134	0.00978
C	3.16801	2.72016	-0.06906
C	4.78739	-1.81808	0.08805
C	2.13997	3.66395	-0.17857
C	2.43625	5.02319	-0.23386
C	3.76198	5.45476	-0.17783
C	4.7902	4.5188	-0.06637
C	4.49621	3.15854	-0.01301
C	6.03579	-1.36599	-0.35936
C	7.13709	-2.21729	-0.36061
C	7.01133	-3.52937	0.09633
C	5.77613	-3.98404	0.55713
C	4.67226	-3.13483	0.55249
H	-7.43392	-1.02167	-0.17984
H	-8.53563	1.13249	0.33654
H	-7.14567	3.17891	0.74756
H	-4.65681	3.02272	0.68543
H	-0.80425	1.27873	0.12122
H	0.14197	-2.95239	0.15125
H	-2.2293	-3.50157	0.10804
H	2.10359	-2.42148	0.07922
H	1.11032	3.31023	-0.22139
H	1.62844	5.75156	-0.32166
H	3.99341	6.52042	-0.22053
H	5.82849	4.8512	-0.01985
H	5.28652	2.41386	0.07685
H	6.11782	-0.33661	-0.70782
H	8.10149	-1.85451	-0.71971
H	7.87574	-4.19519	0.09893
H	5.6727	-5.00413	0.93007
H	3.71911	-3.49687	0.94037

S1 geometry of PXSe2DPm

C	-5.33342	-0.25903	0.01244
C	-4.59049	0.93239	-0.21769
C	-6.74608	-0.20058	-0.01118
C	-7.39995	0.98198	-0.27023
C	-6.65417	2.16885	-0.47986
C	-5.27883	2.14367	-0.4448
O	-3.25983	1.02734	-0.17945
C	-2.37458	0.00441	-0.09138
C	-2.77677	-1.37132	-0.12094
Se	-4.4901	-1.83488	0.52092
C	-1.03425	0.36501	-0.05289
C	-0.03697	-0.61225	-0.06041
C	-0.42726	-1.99486	-0.11692
C	-1.74997	-2.35231	-0.1241
C	1.3724	-0.23272	-0.03698
N	2.28284	-1.22139	-0.06534
C	3.56904	-0.88603	-0.04874
C	3.96812	0.45626	-0.01043
C	2.95221	1.42534	0.02729
N	1.6721	1.07767	0.01199
C	3.26174	2.88241	0.06873
C	4.56009	-1.99815	-0.09235
C	2.27865	3.80771	-0.30542
C	2.54734	5.17288	-0.28331
C	3.79967	5.63557	0.12267
C	4.78056	4.72315	0.50817
C	4.51396	3.35598	0.47986
C	4.15883	-3.26087	-0.54712
C	5.06797	-4.31292	-0.60524
C	6.38942	-4.12203	-0.19994
C	6.79504	-2.87199	0.26536
C	5.8869	-1.81683	0.31738
H	-7.31105	-1.11705	0.17258
H	-8.4888	1.0053	-0.30987
H	-7.1716	3.11024	-0.66634
H	-4.67918	3.04143	-0.59632
H	-0.76536	1.42117	-0.06542
H	0.35688	-2.74929	-0.15225
H	-2.03031	-3.40914	-0.13039
H	5.01667	0.73693	-0.06454
H	1.30485	3.42987	-0.61616
H	1.77513	5.88226	-0.58503
H	4.00953	6.70612	0.14225
H	5.75817	5.07713	0.83866
H	5.28382	2.65539	0.80548
H	3.1222	-3.39364	-0.85612
H	4.74428	-5.28937	-0.969
H	7.10139	-4.94773	-0.24276
H	7.82327	-2.71807	0.59597
H	6.21238	-0.8506	0.70492

T1 geometry of PXSeDRZ

C	5.40137	-0.15322	0.00758
C	4.63782	1.02298	-0.03879
C	6.80461	-0.06513	0.00804
C	7.43397	1.16562	-0.03915
C	6.6649	2.33989	-0.08697
C	5.2846	2.26893	-0.08627
O	3.29059	1.08183	-0.03355
C	2.41418	0.05478	-0.02098
C	2.84045	-1.34244	-0.02761
Se	4.60644	-1.84921	0.07886
C	1.08711	0.38894	-0.01572
C	0.08298	-0.61692	-0.0162
C	0.50538	-2.01964	-0.03006
C	1.81973	-2.35257	-0.0332
C	-1.30528	-0.27026	-0.0095
N	-2.219	-1.2696	-0.01478
C	-3.48886	-0.89629	-0.00894
N	-3.90861	0.37572	0.00109
C	-2.94079	1.30487	0.00544
N	-1.64608	1.04083	0.00096
C	-3.36245	2.73406	0.01638
C	-4.52966	-1.96274	-0.01362
C	-2.40234	3.75273	0.02394
C	-2.79682	5.08734	0.03408
C	-4.15312	5.41665	0.0367
C	-5.11335	4.40501	0.02925
C	-4.72128	3.06861	0.0192
C	-5.8869	-1.62164	-0.00953
C	-6.85959	-2.61824	-0.01375
C	-6.48549	-3.96199	-0.02197
C	-5.1331	-4.30671	-0.02601
C	-4.15896	-3.31256	-0.0219
H	7.39537	-0.98285	0.04427
H	8.52253	1.21899	-0.04
H	7.15416	3.3135	-0.12458
H	4.65859	3.1606	-0.12116
H	0.80203	1.43941	-0.01941
H	-0.27057	-2.78265	-0.03924
H	2.11904	-3.40298	-0.04129

H	-1.34856	3.47541	0.02181
H	-2.04352	5.87669	0.04004
H	-4.46162	6.46344	0.04462
H	-6.1744	4.65921	0.03132
H	-5.4562	2.26428	0.01335
H	-6.15925	-0.56673	-0.003
H	-7.91602	-2.34549	-0.01057
H	-7.24853	-4.74222	-0.02523
H	-4.83747	-5.35699	-0.03238
H	-3.0983	-3.56222	-0.02491

T1 geometry of PXSe4DPm

C	-5.43657	-0.20681	-0.03647
C	-4.65984	0.94918	0.13322
C	-6.8378	-0.10313	-0.04681
C	-7.45589	1.12437	0.11473
C	-6.67498	2.27791	0.28804
C	-5.29503	2.19106	0.29605
O	-3.31188	0.99471	0.13246
C	-2.44851	-0.04511	0.07443
C	-2.90348	-1.43597	0.05784
Se	-4.65484	-1.89685	-0.27752
C	-1.11907	0.26272	0.07129
C	-0.11944	-0.75488	0.0554
C	-0.57749	-2.15014	0.07077
C	-1.89705	-2.46051	0.06379
C	1.26635	-0.39749	0.04304
C	2.31062	-1.35671	0.04372
C	3.62191	-0.89196	0.04112
N	3.89599	0.41829	0.01463
C	2.85537	1.26489	0.00941
N	1.57949	0.92309	0.02883
C	3.1788	2.72254	-0.01899
C	4.78417	-1.82388	0.04301
C	2.15654	3.67497	-0.10573
C	2.46148	5.03301	-0.13533
C	3.79031	5.45484	-0.07613
C	4.81267	4.5103	0.01278
C	4.50993	3.15104	0.0405
C	6.02868	-1.36978	-0.41242
C	7.12835	-2.22312	-0.43293
C	7.0047	-3.53929	0.01259
C	5.77345	-3.99597	0.48164
C	4.67131	-3.14445	0.4964
H	-7.43703	-1.00646	-0.17912
H	-8.54377	1.18975	0.1094
H	-7.15392	3.24896	0.4165
H	-4.6606	3.06809	0.42467
H	-0.81413	1.30689	0.10459
H	0.15015	-2.9591	0.09395
H	-2.21201	-3.50641	0.06705
H	2.10526	-2.4235	0.01802
H	1.12453	3.32838	-0.15131
H	1.6581	5.76819	-0.20549
H	4.02854	6.51963	-0.09882
H	-4.73526	-5.33707	0.67392
H	-7.09642	-4.95677	-0.01876
H	-7.82093	-2.68653	-0.73794
H	-6.20759	-0.81702	-0.7619
H	5.85333	4.83492	0.06169
H	5.29562	2.39961	0.11262
H	6.10933	-0.3371	-0.75144
H	8.08981	-1.85863	-0.79814
H	7.86786	-4.20664	0.00021
H	5.67198	-5.01924	0.84637
H	3.72131	-3.50745	0.89136

T1 geometry of PXSe2DPm

C	5.34287	-0.225	0.00137
C	4.60913	0.95733	-0.17557
C	6.74644	-0.17213	0.00705
C	7.40941	1.03065	-0.1666
C	6.6713	2.21032	-0.34754
C	5.2887	2.17354	-0.3507
O	3.26297	1.05301	-0.16512
C	2.36353	0.04363	-0.11172
C	2.77073	-1.36328	-0.13122
Se	4.49654	-1.88288	0.25989
C	1.04541	0.39794	-0.08753
C	0.02188	-0.59342	-0.08068
C	0.42294	-2.0032	-0.14141
C	1.73011	-2.35759	-0.15642
C	-1.36202	-0.22856	-0.04155
N	-2.27811	-1.22614	-0.05962
C	-3.56001	-0.89325	-0.03018
C	-3.96404	0.45097	0.02435
C	-2.95037	1.42692	0.02803
N	-1.67161	1.09009	0.00045
C	-3.27163	2.88157	0.08558
C	-4.55227	-2.00575	-0.03595
C	-2.29476	3.78657	0.52028
C	-2.57381	5.14806	0.59033
C	-3.83022	5.62743	0.21743
C	-4.80485	4.73575	-0.22763
C	-4.5279	3.37168	-0.29143
C	-4.14983	-3.2903	0.35151
C	-5.05978	-4.3431	0.36199
C	-6.38372	-4.13059	-0.02439
C	-6.79072	-2.85817	-0.42256
C	-5.88141	-1.80245	-0.42664
H	7.31233	-1.09603	0.14426
H	8.49903	1.05557	-0.16549
H	7.18493	3.16213	-0.48581
H	4.68635	3.07218	-0.48448
H	0.77515	1.45239	-0.10398
H	-0.36615	-2.75165	-0.18343
H	2.01384	-3.41184	-0.19464
H	-5.0117	0.72302	0.12311
H	-1.31789	3.39524	0.80397
H	-1.80666	5.84142	0.93866
H	-4.04815	6.69526	0.27027
H	-5.78553	5.10383	-0.5327
H	-5.29254	2.68804	-0.66252
H	-3.11142	-3.4394	0.64707
